# Prediction of MicroRNA and Gene Target in Synovium-Associated Pain of Knee Osteoarthritis Based on Canonical Correlation Analysis

**DOI:** 10.1155/2019/4506876

**Published:** 2019-10-13

**Authors:** Haiming Wang, Yue Hu, Yujie Xie, Li Wang, Jianxiong Wang, Lei Lei, Maomao Huang, Chi Zhang

**Affiliations:** ^1^Department of Rehabilitation Medicine, The First Affiliated Hospital of Zhengzhou University, Zhengzhou, 450000 Henan, China; ^2^Rehabilitation Medicine Department, The Affiliated Hospital of Southwest Medical University, Luzhou, Sichuan, China; ^3^Rehabilitation Medicine, Southwest Medical University, Luzhou, Sichuan, China

## Abstract

Inflammation plays a central role in knee osteoarthritis (OA) pathogenesis (C. R. Scanzello, 2017). The synovial membrane inflammation is associated with disease progression and represents a primary source of agony in knee OA (L. A. Stoppiello et al., 2014). Many inflammatory mediators may have biomarker utility. To identify synovium related to knee OA pain biomarkers, we used canonical correlation analysis to analyze the miRNA-mRNA dual expression profiling data and extracted the miRNAs and mRNAs. After identifying miRNAs and mRNAs, we built an interaction network by integrating miRWalk2.0. Then, we extended the network by increasing miRNA-mRNA pairs and identified five miRNAs and four genes (TGFBR2, DST, TBXAS1, and FHLI) through the Spearman rank correlation test. For miRNAs involved in the network, we further performed the Gene Ontology (GO) and the Kyoto Encyclopedia of Genes and Genomes (KEGG) functional enrichment analyses, whereafter only those mRNAs overlapped with the Online Mendelian Inheritance in Man (OMIM) genetic database were analyzed. Receiver operating characteristic (ROC) curve and support vector machine (SVM) classification were taken into the analysis. The results demonstrated that all the recognized miRNAs and their gene targets in the network might be potential biomarkers for synovial-associated pain in knee OA. This study predicts the underlying risk biomarkers of synovium pain in knee OA.

## 1. Introduction

Pain is the main presenting symptom of knee osteoarthritis (OA) patients [3], but the mechanism is rarely known. Synovial inflammation is a major cause of pain in patients with knee OA [4]. Increasing evidence suggested that synovial inflammation plays a significant role in knee OA [5]. Synovium tissue in knee OA is regarded as a treatment target in symptomatic knee OA [6]. Synovial inflammation is promoted by some important mediators [7], and a study has identified some candidate genes associated with synovium pain in knee OA patients [8]. It is crucial to explore the pathogenesis of knee OA from theses biomarker.

Recent studies have established that microRNAs (miRNAs) are mediators of knee OA, which were responsible for the development and progression of OA. For example, circulating miRNA (23a-3p, 24-3p, 27a-3p, 27b-3p, 29c-3p, 34a-5p, and 186-5p) in synovial fluid (SF) has been identified as a potential predictor for knee OA [9]. Bijlsma and Knahr found that an intra-articular injection of miRNA-140 could alleviate knee OA progression by regulating extracellular matrix in rats [10]. Moreover, interacting levels of miRNA targeted by multiple miRNAs have demonstrated the altered expression of genes in knee OA [11]. Notably, targeting miRNA-mRNA interactions, such as miR-140 with ADAMTS5, MMP13, and IGFBP5 have a great influence on OA pathophysiology [12]. However, limited studies have reported the combinatorial analysis of miRNA-mRNA interaction based on miRNA-mRNA expression profiles in knee OA patients related to synovium pain. This study is dedicated to finding the potential risk biomarkers and predictors of synovium OA pain.

## 2. Materials and Methods

### 2.1. Data Source

In the current study, we used miRNA-mRNA dual expression profiling data (GSE99662) to implement our analysis. miRNAs were profiled in knee synovium tissues from knee OA patients with high knee pain (*n* = 5) and low knee pain (*n* = 5) by the next generation of sequencing from the study of Sprott H (Table 1). A number of five female normal knee synovium tissues provided miRNAs from the results of Lotz and coworkers [13]. The processing of raw miRNA and mRNA sequencing data was modified according to our in-house analysis pipeline. Significant differential expression mRNAs and miRNAs between patients and normal tissues were distinguished by the DESeq R package [14]. More rigorous criteria, *p* < 0.05 as the cutoff, was used to filter differentially expressed miRNAs and mRNAs. As a result, 2485 differentially expressed mRNAs and 36 differentially expressed miRNAs were identified for further analysis.

### 2.2. Canonical Correlation Analysis (CCA)

CCA was performed to identify and measure the correlation between two sets of variables to find the association matrix between variables. Notably, if there were too many variables in the analysis, it would be inappropriate to use this analysis method. Only differential expression microRNA and mRNA were selected for target gene prediction, which was achieved by mirWalk2 [15]. In this analysis, miRNAs and mRNAs were taken as the first and second set of variables, respectively. Then, we performed RCC to search for potential associations between miRNAs and mRNAs. The leave-one-out criterion was applied before performing RCC on two sets of variables. R software package (http://www.r-Project.org) was used for CCA.

### 2.3. Network Construction

For the miRNAs and mRNAs, extracted from the CCA, we extended the network of miRNA and mRNA based on retaining the miRNA-targeting relationship obtained from the three miRNA-target sources. According to the Spearman correlation coefficient, we chose only the miRNA-mRNA interaction with *p* value <0.05 to be significant. In this analysis, there were two reasons why multiple tests were not performed. First, it enabled us to retain a high degree of confidence in the interaction network building. Second, it avoided the loss of information. The constructed network predicted miRNA-targeting relationships that might be related to synovium-associated pain with KOA.

### 2.4. GO and KEGG Function Enrichment Analysis

For the five miRNAs in the constructed network, we performed Gene Ontology (GO) function and KEGG enrichment analysis on its target genes. ClusterProfiler software R package (http://www.r-project.org) was used for the analysis. Multiple test corrections were performed based on the Benjamini–Hochberg method [16]. A GO and KEGG term with *p* < 0.05 was considered to be significant.

### 2.5. Verification of Potential Genes Related to Synovium Pain in Knee OA in the Network

#### 2.5.1. Classification Performance Analysis of Potential Synovial-Associated Pain in Knee OA Related Genes Identified

All the ten patients were female, aged 62 to 78 years. OA pain severity status of patients was assessed a day before surgery by a clinical investigator using a 10-point VAS. VAS score of 0–3 indicates low pain and 7–10 high pain. Five patients with high pain, five patients with low pain, and no healthy donors were enrolled. The Local Ethical Committee (KEK-ZH-Nr. 2013-0210) approved the study. Normal human knee cartilage tissues were procured by tissue banks (approved by Scripps Institutional Review Board) from five women. All patients have written informed consent. All experiments were performed in accordance with relevant guidelines and regulations. Standardized surgical excision (excision of the capsule with synovia in the recesses suprapatellar) was performed. The biopsies (two per patient with a size of approximately 2 cm^2^) were fresh frozen (dry ice). RNA was extracted randomly from one biopsy using TRIzol® Reagent (Thermo Fisher Scientific, Waltham, MA, USA). Then, RNA was analyzed by Qubit® 1.0 Fluorometer (Thermo Fisher Scientific) and Agilent 2200 Tape Station (Agilent Technologies, Santa Clara, CA, USA). At last, RNA with RIN (RNA Integrity Number) values 8.3–9.1 was used for sequencing.

To find out whether the genes in the constructed network is related to synovitis-caused knee pain, we manually chose genes overlapping with online Mendelian Inheritance in Man (OMIM) genetic database [17]. The area under the ROC of overlapping genes was calculated to detect target genes. The expression of these overlapping genes was used to distinguish low pain and high pain by the support vector machine (SVM) method [18]. Besides, cross validation could limit problems such as overfitting to obtain more accurate model prediction performance estimates. Because there were not enough data to divide into “training set” and “test set”, it may affect important modeling or testing capabilities. To solve this problem, we carried out cross validation five times in the SVM program. So we divided all the samples into five groups. For each analysis, one group served as test data and the rest as train data.

#### 2.5.2. Cluster Analysis of miRNA and mRNA Expression Profiles to Validate Possible Genes Related to Synovium Pain in Knee OA

**Figure 1:**
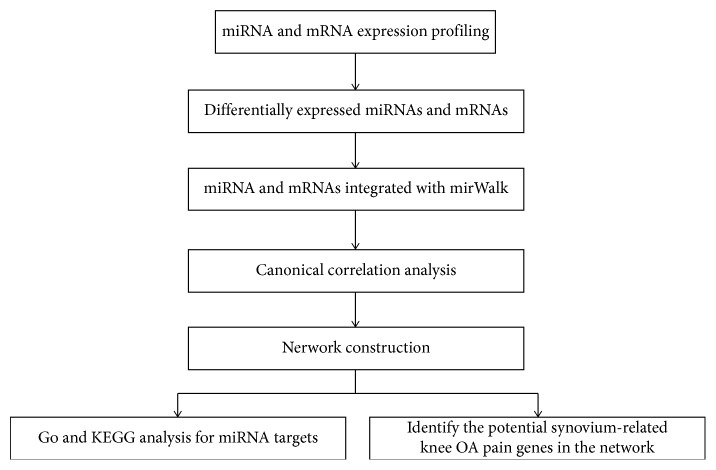
Flow chart of our work.

To verify the potential genes involved in the OA-related network, we combined miRNA with the mRNA expression profiles of 10 knee OA patients and used the similar network fusion (SNF) method in the cluster analysis [19]. SNF gathered the similarities between different data types of knee OA patients through a cross-network diffusion process to build the similarity matrix of the fusion patient. Low-pain and high-pain patients with knee OA were divided into clusters, which intended to compare with normal female synovium tissue. The most significant differentially expressed genes between the clusters and the normal samples were extracted.  Figure 1 illustrates our workflow flow chart for this analysis.

## 3. Results

### 3.1. CCA Results

A total of 6 differentially expressed miRNAs and 425 differentially expressed mRNAs were analyzed by CCA. Because the number of genes was larger than the number of samples, we took the CCA to identify miRNA-mRNA associations from OA synovium pain. Optimal values of two regularization parameters were 0.75 and 0.25 based on the leave-one-out criterion. We obtained four matrixes through regularized canonical correlation: the correlation with miRNA-miRNA canonical variables; the correlation with mRNA-miRNA canonical variables; the correlation with miRNA-mRNA canonical variables; and the correlation with mRNA-mRNA canonical variables. For these four matrixes, we selected the correlation coefficients of the original variable and the first canonical variable because the first canonical variable had the highest canonical correlation coefficient of 0.954 ([Supplementary-material supplementary-material-1]). We identified 5 miRNAs (miR-133a-3p, miR-145-5p, miR-335-5p, miR-224-5p, and miR-215-5p) in Table 1 and 284 mRNAs, 251 genes, and 260 miRNA-mRNA target pairs with correlation coefficients >0.3 (Appendixes [Supplementary-material supplementary-material-1] and [Supplementary-material supplementary-material-1]). These miRNAs and mRNAs were further used for network construction.

### 3.2. Network Construction

**Figure 2:**
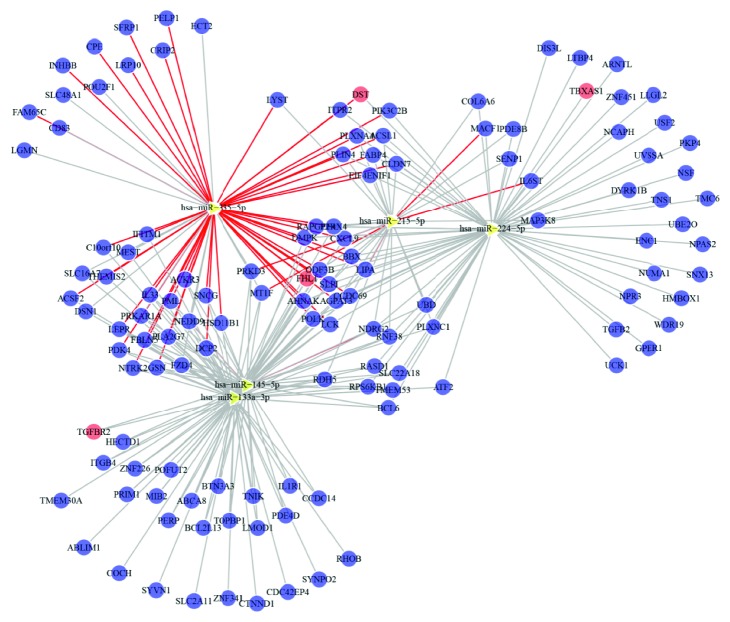
CCA of the selected miRNAs and mRNAs. Spearman correlation was performed according to the expression quantity. All of the miRNA-targets in the figure were much associated. The yellow triangles represent miRNAs, and the circles represent genes. The red lines indicate the experimentally confirmed miRNA-targets in miRWalk2.0 database, while the gray lines only represent the significantly related miRNA-targets. CCA: canonical correlation analysis.

The Spearman rank correlation test was used to analyze 13 miRNAs and 26 mRNAs, which were identified by CCA (Appendix 4). Based on the criterion, *p* < 0.05, we found five miRNAs and 129 genes in the network (Figure 2). From the constructed network, we found that some target genes of miRNAs were associated with synovium OA pain. For example, leptin receptor (LEPR) gene has been found in the network and associated with susceptibility to knee OA [20]. Interesting, our network found that miR-335-5p could regulate LEPR. Transforming growth factor-b type II receptor (TGFBR2) has found to influence cartilage metablism [21], which can be regulated by miR-133a-3p, miR-145-5p, and miR-224-5p.

### 3.3. Gene Ontology (GO) Function Enrichment Analysis

**Figure 3:**
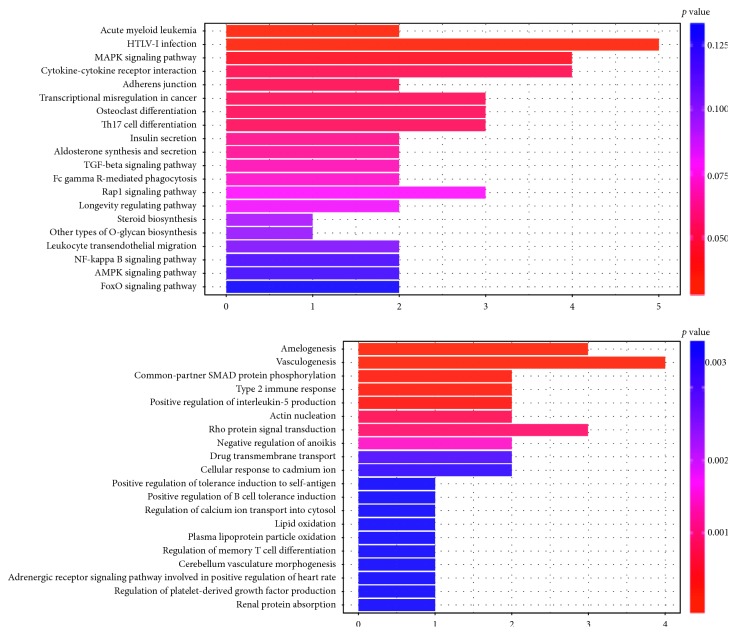
One of the miRNA enrichment analyses of target genes networks. Significant (a) KEGG and (b) GO terms of the biological process are presented in different colors. OA: osteoarthritis; GO: gene ontology; KEGG: Kyoto Encyclopedia of Genes and Genomes.

GO enrichment analysis was performed for each miRNA target gene in the network.  Figure 3 shows GO and KEGG function enrichment analysis of target mRNA of miR-133a-3p. Cooler colors indicate more significant GO terms (the *p* values displayed in the graph were not adjusted). As shown in Figure 3, the target genes of miR-133a-3p were enriched on vasculogenesis by GO analysis, MAPK signaling pathway cytokine, or receptor interaction by KEGG analysis.

### 3.4. Classification Performance Analysis of Genes with Potential Synovium-Related OA Pain

**Figure 4:**
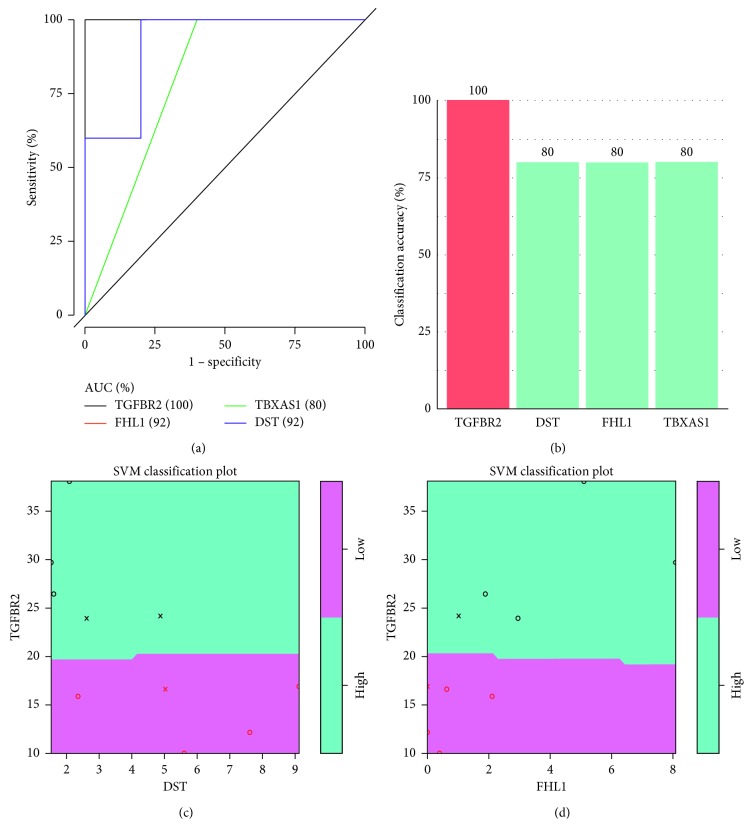
Classification performance analysis of four genes overlapped with synovial pain knee OA genes identified in the OMIM database. (a) ROC analysis of the four genes: TGFBR2, DST, TBXAS1, and FHL1. AUC was above 0.8. Classification accuracy was above 80%. (b) Classification accuracy of the four genes based on the SVM method. (c, d) The SVM classification diagram of TGFBR2, DST, TBXAS1, and FHL1. OMIM: Online Mendelian Inheritance in Man; ROC: receiver operating characteristic curve; AUC: area under the Curve; SVM: support vector machine.

We manually chose genes overlapping with synovial-associated pain in KOA-related genes in online database OMIM. Four important genes (TGFBR2, DST, TBXAS1, FHLI) have been found to be associated with synovium-related OA pain and the area under the curve (AUC) more than 0.8 (Figure 4(a)). The expression of these four genes is considered as the prediction. The classification accuracy rate of TGFBR2, DST, TBXAS1, and FHLI, respectively, was more significant than 0.8. TGFBR2 showed higher classification accuracy rate than other three genes based on SVM classification plot (Figure 4(b)). DST and FHL1 showed excellent classification performance in low pain and were distinguished well from high-pain patients (Figures 4(c) and 4(d)).

### 3.5. Cluster Analysis of miRNA and mRNA Expression Profiles to Validate Possible Genes Involved Synovial-Related Knee OA Pain

**Figure 5:**
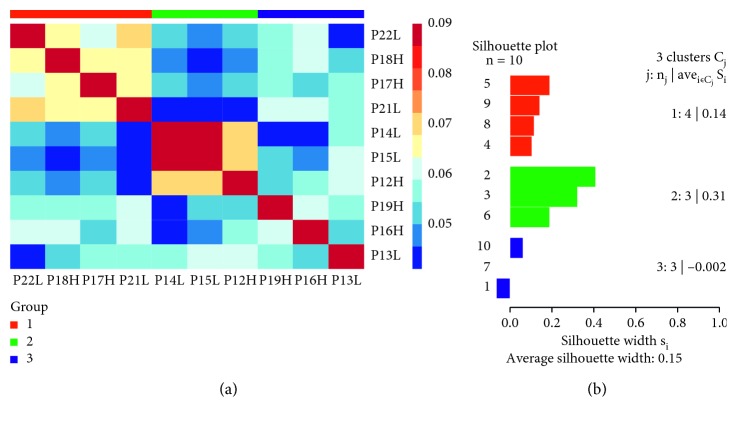
(a) miRNA and mRNA expression profiles of 10 knee OA patients clustered based on the SNF method. The graph was a thermal map of cluster analysis. (b) 3 cluster samples were compared with the normal samples at the mRNA level for differential gene analysis. The silhouette score represented the consistency within the data cluster. OA: osteoarthritis; SNF: similar network fusion.

SNF results suggested that low-pain and high-pain patients with knee OA be divided into three clusters and then compared with five normal female synovium tissues individually. A heat map in Figure 5(a) and a silhouette plot map in Figure 5(b) described the three clusters. Cluster 1 included four patients (P22L, P18H, P17H, and P21L). Cluster 2 included three patients (P14L, P15L, and P12H), and cluster 3 included three patients (P19H, P16H, and P13L). In this analysis, the silhouette score of cluster 2 was the highest (S1 = 0.31), which suggested a greater similarity among samples of the same cluster. We extracted 400 significant differentially expressed genes from the 3 clusters and the normal samples for each of the three clusters. Surprisingly, DST and TBXAS1 were the common genes that existed in the compartment results of all the individual clusters vs. normal. Hence, these hub genes in the network could be considered as potential synovium-related OA pain genes.

## 4. Discussion

This study predicted potential biomarkers of synovial pain in knee OA. At present, it has proved that age, sex, and body weight are risk factors for knee OA [22]. By performing integration data analysis, we can find the potential biomarkers of synovium-associated knee OA pain. Previous researches have shown that the metastasis of cancer was related to the abnormal regulation of miRNA-mRNA [23]. There are few studies to prove the relationship between miRNA and their targets in synovium-associated knee OA pain. Meanwhile, few studies have reported a correlation between miRNA-mRNA maladjustment and synovium-associated pain in knee OA. The reason might be that there is poor data availability on pre-double expression of miRNA-mRNA.

In this study, we performed CCA to find potential biomarkers of synovium-associated knee OA pain. miRNA-mRNA coexpression data were analyzed to ascertain the correlation between miRNA-mRNA and synovial pain in knee OA. CCA applies a nonlinear function to the original variables and extracts related components from two sets of variables [24]. CCA does not require a dataset to have the same dimension and can be used for more than two datasets at the same time. On the other hand, CCA does not assume the directionality of the relationships between datasets. That is contrary to specifying the regression method for both independent and dependent datasets. Besides, CCA not only quantifies the similarity between datasets, but also describes relationships between datasets in an interpretable way [25]. Meanwhile, GO enrichment analysis was performed on target genes in this research. GO is commonly used in the biomedical research community for analysis-histology and related data. It describes functions from three aspects: molecular functions, cellular components, and biological processes [26].

Our research found that TGFBR2, DST, TBXAS1, and FHL1 genes were closely linked to susceptibility to synovial pain in patients with knee OA. TGFBR2 gene can promote the bone formation of osteoblasts and affect the maintenance of bone mass homeostasis. Knockout of TGFBR2 will reduce the severity of OA [27]. Dystonin is a cytoskeletal crosslinker protein that binds to actin and tubulin. This protein dysfunction can lead to sensory neuropathy, known as the human muscle-atrophic sensory autonomic neuropathy [28]. According to our research, this is the first time that dystonin and synovium pain in KOA have correlated. The relationship between the two needs further study. Then, TbXAS1 is a member of cytochrome P450 superfamily, which can directly or indirectly provide angiogenic factors to platelets to promote angiogenesis. TBXAS1 can catalyze the conversion of prostaglandin H2 to thromboxane A2 and then induce platelet aggregation and smooth muscle contraction [29]. FHL1 gene is highly expressed in skeletal muscle and participates in the growth and differentiation of muscle cells. It may regulate cell functions mediated by integrins, such as cytoskeletal rearrangement [30]. We took the expression of these four genes as prediction variables and used a support vector machine to classify the samples. The classification accuracy of TGFBR2, DST, TBXAS1, and FHL1 was 100%, 92%, 80%, and 92%, respectively (Figure 4(a)). Among them, TGFBR2 showed a classification accuracy of 100%. Therefore, we used the SVM classification map to determine the classification performance of the three genes (TGFBR2, DST, and FHL1), which showed a higher classification accuracy than TBXAS1.

In this construction network, we observed five newly identified miRNAs has-miR-133a-3p, has-miR-145-5p, has-miR-335-5p, has-miR-224-5p, and has-miR-215-5p, which can regulate synovial pain in knee OA. Has-miR-133a-3p was involved in osteoclast formation, differentiation, apoptosis, and resorption, showing great promise as a potential therapeutic target for biomarkers and osteoporosis [31], and was found to regulate TGFBR2 and FHL1 expression in this study. Surprisingly, has-miR-145-5p was also detected to regulate TGFBR2 and FHL1, which has been discovered to be associated with changes in cartilage matrix production and degradation levels in patients with OA [32]. Furthermore, has-miR-335-5p can regulate DST and FHL1. Previous studies have shown that has-miR-335-5p expression is elevated in OA patients. MiR-335-5p can regulate the differentiation process of hMSCs chondrocytes induced by transforming growth factor *β*3. SoMiR-335-5p has a potential role in regulating genes to maintain more progenitor phenotypes [33]. Furthermore, has-miR-224-5p plays a significant role in the pathogenesis of femoral head necrosis and may be a potential biomarker for the prognosis of it [34]. Interestingly, we found that this microRNA also regulated the expression of DST, TBXAS1, and FHL1, suggesting that it may be related to synovial pain in KOA.

Meanwhile, there has been no evidence of a link between has-miR-215-5p and KOA. In this research, we studied that has-miR-215-5p regulated the expression of FHL1. However, these miRNA target relationships obtained require further validation in molecular biology experiments.

SNF shows its ability to integrate data when using different types of data measurements at the same time [35]. Fortunately, we used the SNF analysis to find that DST and TBXAS1 genes exist in all 3 clusters in our study. These results suggested that the two genes may play a key role in the progress of synovium pain in knee OA.

There are three limitations in our study. First, we have selected only verified targeted genes by experiment in the database. Some predicted miRNA target regulation had not been incorporated into the analysis. Second, some exciting findings were based on a small sample. We plan to increase the sample in the next study. Third, synovial fluid exosomal miRNA content has been found altered in patients with OA, and these changes were gender-specific [36]. The samples' data we used were of only female, so the research result cannot be translated into a general OA population, especially male. The research data should be interpreted carefully.

These predictive analyses revealed that some miRNAs associated with knee OA were involved in synovium pain-related genes, especially DST and TBXAS1. Therefore, our consequences can be used as a reference for future explorers on the pathogenesis and genetic causes of synovial pain in knee OA. In future studies, it is necessary to explore the potential mechanism of controlling complex molecular regulation by integrating multiple data types. These studies will be helpful for further experimental verification and discovery of knee OA biomarkers.

## Figures and Tables

**Table 1:** According to the next generation of sequencing from the study of Sprott H, the miRNAs were profiled in knee synovium tissues from knee OA patients with high knee pain (*n* = 5) and low knee pain (*n* = 5).

miRNA	Correlation_coefficient
hsa-miR-133a-3p	0.8568404
hsa-miR-145-5p	0.8988057
hsa-miR-215-5p	0.825922
hsa-miR-224-5p	0.7471897
hsa-miR-335-5p	0.8872083

## Data Availability

Most of the data used to support the findings of this study are included within the supplementary information files. Other accurate data could available from the corresponding author upon request.

## Supplementary Materials

Supplementary Materials Appendix 1: four matrixes. We selected the correlation coefficients of the original variable and the first canonical variable because the first canonical variable had the highest canonical correlation coefficient of 0.954. Appendixes 2 and 3: the miRNAs and mRNAs selected in this study, which will be further used in network construction. Appendix 4: 13 miRNAs and 26 mRNAs identified by CCA, which were analyzed by the Spearman rank correlation test.
