# Supplementary material for: Prediction of MicroRNA and Gene Target in Synovium-Associated Pain of Knee Osteoarthritis Based on Canonical Correlation Analysis
Source: Biomed Res Int. 2019 Oct 13;2019:4506876. doi: 10.1155/2019/4506876 (PMC6815580; doi:10.1155/2019/4506876)
Supplement: Supplementary Materials — Appendix 1: four matrixes. We selected the correlation coefficients of the original variable and the first canonical variable because the first canonical variable had the highest canonical correlation coefficient of 0.954. Appendixes 2 and 3: the miRNAs and mRNAs selected in this study, which will be further used in network construction. Appendix 4: 13 miRNAs and 26 mRNAs identified by CCA, which were analyzed by the Spearman rank correlation test. [file 4506876.f1.zip › 4506876.f1/appendix 2_BMRI_2859734.docx]

| Appendix 2 | | | |
| --- | --- | --- | --- |
| GeneName | Correlation_coefficient | GeneName | Correlation_coefficient |
| ABCA8 | 0.8022328 | MROH1 | 0.472039 |
| ABLIM1 | 0.7259898 | MT1F | 0.7100108 |
| ACE | 0.313275 | MYO18A | 0.4215441 |
| ACKR3 | 0.7832325 | MYO5A | 0.3638906 |
| ACSF2 | 0.5690572 | NBEA | 0.442383 |
| ACSL1 | 0.8543091 | NCAPH | 0.4867247 |
| AGPAT3 | 0.8405216 | NCEH1 | 0.3491905 |
| AHNAK | 0.7790128 | NDRG2 | 0.7841674 |
| ANK2 | 0.3888941 | NEDD9 | 0.6658751 |
| ANKRD6 | 0.4279813 | NPAS2 | 0.6525648 |
| AP1G1 | 0.5633578 | NPR3 | 0.5299703 |
| ARHGEF28 | 0.3764602 | NSF | 0.7060405 |
| ARNTL | 0.650007 | NTN4 | 0.4684299 |
| ATF2 | 0.593906 | NTRK2 | 0.8570888 |
| ATP7A | 0.4882234 | NUMA1 | 0.5339404 |
| BBX | 0.7579714 | ODF3B | 0.8477502 |
| BCL2L1 | 0.5453479 | OGDH | 0.4867036 |
| BCL2L13 | 0.623708 | P2RX4 | 0.5933073 |
| BCL6 | 0.8138827 | PABPC4 | 0.3866373 |
| BPTF | 0.3329874 | PARN | 0.3246128 |
| BTN3A3 | 0.5493814 | PARP8 | 0.4756223 |
| C10orf10 | 0.8349221 | PDE4A | 0.5626823 |
| CCDC14 | 0.4303305 | PDE4D | 0.634651 |
| CCDC69 | 0.9071132 | PDE5A | 0.5771344 |
| CD83 | 0.6911189 | PDE8B | 0.7300535 |
| CDC42EP4 | 0.6906888 | PDK4 | 0.9317867 |
| CERCAM | 0.497213 | PDLIM3 | 0.5088405 |
| CHI3L2 | 0.5094388 | PDZD2 | 0.5988604 |
| CHL1 | 0.3765817 | PEBP1 | 0.4011082 |
| CLDN7 | 0.661142 | PELP1 | 0.6555188 |
| COBLL1 | 0.3537849 | PERP | 0.761785 |
| COCH | 0.7725436 | PIK3C2B | 0.6558355 |
| COL6A6 | 0.5229311 | PKM | 0.418615 |
| COLGALT2 | 0.5565345 | PKP4 | 0.463132 |
| CPE | 0.6433352 | PLA2G7 | 0.6148504 |
| CPT1B | 0.3302312 | PLAT | 0.4017456 |
| CRIP2 | 0.628536 | PLD3 | 0.3598889 |
| CSF1 | 0.4326964 | PLIN4 | 0.85754 |
| CTNND1 | 0.641407 | PLSCR4 | 0.656084 |
| CXCL9 | 0.586646 | PLXNA4 | 0.8104353 |
| DCP2 | 0.650089 | PLXNC1 | 0.6520877 |
| DICER1 | 0.345017 | PML | 0.7527086 |
| DIS3L | 0.5095395 | PODXL | 0.3060532 |
| DMPK | 0.5518614 | POFUT2 | 0.4082271 |
| DNAJC19 | 0.4166224 | POLK | 0.8173657 |
| DSN1 | 0.7532665 | POU2F1 | 0.5507584 |
| DST | 0.7462663 | PPARA | 0.5026916 |
| DYRK1A | 0.3986288 | PPM1L | 0.4923022 |
| DYRK1B | 0.6321139 | PPP1R26 | 0.5484728 |
| EBF1 | 0.4691631 | PRIM1 | 0.6076625 |
| ECT2 | 0.5735222 | PRKAR1A | 0.8030891 |
| EDEM3 | 0.5361102 | PRKD3 | 0.6813139 |
| EIF4ENIF1 | 0.6890094 | PTBP2 | 0.5366081 |
| ENC1 | 0.5299465 | PTP4A2 | 0.3864497 |
| EXD2 | 0.3395543 | RAPGEF4 | 0.8089862 |
| FABP4 | 0.8368779 | RARRES2 | 0.6712781 |
| FADS2 | 0.5021278 | RASD1 | 0.6863656 |
| FAM135A | 0.4966302 | RDH5 | 0.7731705 |
| FAM3B | 0.345027 | RHOB | 0.4092673 |
| FAM45A | 0.3311557 | RNF214 | 0.5844006 |
| FAM65C | 0.7379072 | RNF38 | 0.7529066 |
| FBLN5 | 0.8202209 | RPS6KB1 | 0.6911824 |
| FBN1 | 0.607915 | RRBP1 | 0.3889367 |
| FBXO25 | 0.5987472 | SAT1 | 0.6221536 |
| FHL1 | 0.8882017 | SCIN | 0.5160718 |
| FMR1 | 0.406035 | SENP1 | 0.6477884 |
| FREM1 | 0.6476354 | SERPINB1 | 0.516782 |
| FZD4 | 0.8654306 | SFRP1 | 0.7079605 |
| GABARAPL1 | 0.3978786 | SH2D3C | 0.5009065 |
| GFOD1 | 0.4937473 | SLC16A7 | 0.9198912 |
| GLG1 | 0.3952138 | SLC22A18 | 0.6998743 |
| GPER1 | 0.6042908 | SLC26A7 | 0.4566078 |
| GSN | 0.6707247 | SLC2A11 | 0.6434084 |
| H6PD | 0.4366619 | SLC37A1 | 0.4133875 |
| HDAC6 | 0.4534942 | SLC48A1 | 0.7135761 |
| HECTD1 | 0.5994644 | SLPI | 0.8528271 |
| HEY1 | 0.5092436 | SMAD4 | 0.370956 |
| HLA-DRB5 | 0.4726712 | SMC4 | 0.6934332 |
| HMBOX1 | 0.6093781 | SMG7 | 0.3867045 |
| HMGA1 | 0.3211733 | SNCG | 0.8673259 |
| HSD11B1 | 0.9127465 | SNED1 | 0.4642305 |
| HSD17B7 | 0.3218087 | SNX10 | 0.3618659 |
| HSPG2 | 0.3198265 | SNX13 | 0.5054727 |
| IFITM1 | 0.7055411 | SORBS2 | 0.4298349 |
| IGFBP4 | 0.602099 | ST3GAL5 | 0.5845861 |
| IKBKB | 0.383855 | SYNPO2 | 0.5976976 |
| IL1R1 | 0.5505293 | SYVN1 | 0.5528704 |
| IL33 | 0.7416881 | TACC2 | 0.6059988 |
| IL6ST | 0.6195971 | TBL1X | 0.4058531 |
| INHBB | 0.8053942 | TBXAS1 | 0.5302803 |
| IQGAP1 | 0.6626672 | TCHP | 0.4692716 |
| IRAK4 | 0.3943439 | TEAD2 | 0.6608582 |
| ITGB2 | 0.5116483 | TGFB2 | 0.549233 |
| ITGB4 | 0.6607229 | TGFBR2 | 0.8421356 |
| ITPR2 | 0.6239042 | THEMIS2 | 0.7002097 |
| KIAA1324L | 0.3728998 | TMC6 | 0.3964585 |
| KLF7 | 0.3029989 | TMEM30A | 0.6604534 |
| LBP | 0.5538265 | TMEM53 | 0.7121907 |
| LCA5 | 0.4108177 | TNIK | 0.6931748 |
| LCK | 0.7326405 | TNS1 | 0.452621 |
| LCOR | 0.5579798 | TOPBP1 | 0.5954934 |
| LEPR | 0.837368 | TPM1 | 0.6694976 |
| LGMN | 0.6164389 | TPM3 | 0.3241415 |
| LHX6 | 0.5010103 | TRIM16 | 0.3604296 |
| LIPA | 0.8420222 | TRIM2 | 0.4847016 |
| LLGL2 | 0.4949804 | TSC22D3 | 0.3662753 |
| LMO3 | 0.3130483 | TTLL1 | 0.3515817 |
| LMOD1 | 0.728434 | TULP3 | 0.5432247 |
| LRP10 | 0.6693818 | UACA | 0.4074067 |
| LRRFIP2 | 0.3443537 | UBD | 0.6439229 |
| LTBP4 | 0.5919481 | UBE2O | 0.5424422 |
| LYST | 0.6872054 | UBN1 | 0.4687191 |
| MACF1 | 0.5523804 | UCK1 | 0.7654246 |
| MAFF | 0.5271072 | USF2 | 0.4088352 |
| MAP2 | 0.5800298 | USP54 | 0.4408674 |
| MAP3K8 | 0.8261055 | UVSSA | 0.4188261 |
| MCAM | 0.3751794 | VEGFA | 0.4073992 |
| MDM4 | 0.7054657 | WDR19 | 0.3001113 |
| MEST | 0.7275179 | WISP3 | 0.4832169 |
| MFGE8 | 0.4107524 | ZEB1 | 0.337093 |
| MGA | 0.4729613 | ZFP1 | 0.6195683 |
| MIB2 | 0.7581924 | ZNF226 | 0.550663 |
| MIS18BP1 | 0.5423515 | ZNF273 | 0.5538061 |
| ZNF451 | 0.5734315 | ZNF280D | 0.6065475 |
| ZNF461 | 0.4846389 | ZNF341 | 0.5505299 |
| ZNF611 | 0.4499032 |  |  |
