# Supplementary material for: Prediction of MicroRNA and Gene Target in Synovium-Associated Pain of Knee Osteoarthritis Based on Canonical Correlation Analysis
Source: Biomed Res Int. 2019 Oct 13;2019:4506876. doi: 10.1155/2019/4506876 (PMC6815580; doi:10.1155/2019/4506876)
Supplement: Supplementary Materials — Appendix 1: four matrixes. We selected the correlation coefficients of the original variable and the first canonical variable because the first canonical variable had the highest canonical correlation coefficient of 0.954. Appendixes 2 and 3: the miRNAs and mRNAs selected in this study, which will be further used in network construction. Appendix 4: 13 miRNAs and 26 mRNAs identified by CCA, which were analyzed by the Spearman rank correlation test. [file 4506876.f1.zip › 4506876.f1/appendix 3_BMRI_2859736.docx]

| Appendix 3 | | | | | | | | | | |
| --- | --- | --- | --- | --- | --- | --- | --- | --- | --- | --- |
|  | P13L | P14L | P15L | P21L | P22L | P12H | P16H | P17H | P18H | P19H |
| hsa-miR-133a-3p | 7.56 | 0.5 | 1.56 | 5.77 | 7.91 | 12.56 | 21.64 | 11.03 | 8.24 | 3.49 |
| hsa-miR-145-5p | 807.8 | 434.21 | 412.41 | 617.73 | 848.43 | 1504.5 | 1939.54 | 1139.38 | 801.32 | 654.71 |
| hsa-miR-215-5p | 6.1 | 7.69 | 5.56 | 4.4 | 11.04 | 15.44 | 72.61 | 3.89 | 12.15 | 5.96 |
| hsa-miR-224-5p | 45.11 | 91.62 | 49.56 | 37.2 | 76.96 | 163.75 | 200.83 | 57.53 | 85.25 | 81.97 |
| hsa-miR-335-5p | 117.56 | 113.02 | 73.37 | 538.62 | 689.51 | 586.8 | 1017.1 | 251.11 | 739.28 | 425.53 |
| ENST00000489635 | 0 | 0 | 0 | 0 | 0.33 | 0.64 | 0.68 | 0.34 | 0 | 0.37 |
| ENST00000602477 | 1.19 | 0.39 | 0.36 | 0 | 0.25 | 0.89 | 1.17 | 0.89 | 1.09 | 0.64 |
| ENST00000412328 | 0 | 3.47 | 2.1 | 0 | 0 | 5.96 | 8.61 | 7.88 | 6.14 | 11.98 |
| ENST00000373925 | 0 | 0 | 0 | 0.75 | 0.4 | 0.29 | 1.87 | 1.21 | 1.82 | 0 |
| ENST00000468531 | 0.92 | 0.78 | 2.62 | 0.58 | 5.54 | 4.11 | 2.42 | 7.3 | 7.73 | 3.93 |
| ENST00000333070 | 0.5 | 0.24 | 0.61 | 0.26 | 0 | 0 | 0 | 0 | 0 | 0 |
| ENST00000361689 | 3.13 | 0.61 | 0.73 | 2.18 | 0 | 0 | 0 | 0.92 | 0.15 | 0 |
| ENST00000437136 | 2.49 | 1.39 | 1.5 | 1.26 | 3.09 | 0.4 | 0 | 0 | 1.45 | 0 |
| ENST00000372243 | 0 | 0 | 0 | 0 | 0 | 0.62 | 0.34 | 0.25 | 0.32 | 0 |
| ENST00000372244 | 0.53 | 0.24 | 0.45 | 0.19 | 0.51 | 0 | 0 | 0.07 | 0 | 0 |
| ENST00000371060 | 2.86 | 1.73 | 2.34 | 5.33 | 2.86 | 5.59 | 8.82 | 4.7 | 6.84 | 3.01 |
| ENST00000462765 | 0.92 | 0.25 | 0.27 | 0 | 0.57 | 0.5 | 2.19 | 1.22 | 1.18 | 0.3 |
| ENST00000426398 | 1.22 | 1.21 | 0 | 0.81 | 0.91 | 0.66 | 2.7 | 2.73 | 2.91 | 0.77 |
| ENST00000488198 | 1.84 | 0.95 | 0 | 0 | 0 | 0 | 0 | 0 | 0 | 0 |
| ENST00000477185 | 0 | 0 | 0 | 0 | 0 | 0 | 2.21 | 0 | 0 | 1.45 |
| ENST00000368545 | 0.7 | 0 | 0 | 0 | 0.03 | 0.62 | 0.55 | 0 | 0.59 | 1.61 |
| ENST00000254521 | 1.49 | 0.7 | 0 | 0 | 0.47 | 0 | 0 | 0 | 0.27 | 0.04 |
| ENST00000367865 | 0 | 0.17 | 0.12 | 0.68 | 0.12 | 0.34 | 0.75 | 0.42 | 0.91 | 0.62 |
| ENST00000367537 | 0 | 0 | 0 | 0 | 0 | 0 | 0.43 | 0.13 | 0 | 0.51 |
| ENST00000367521 | 0.09 | 0.22 | 0.06 | 0.19 | 0.14 | 0.04 | 1.26 | 0.69 | 0.37 | 0.5 |
| ENST00000466392 | 2.56 | 0.76 | 1.14 | 2.47 | 0.13 | 0.43 | 0 | 0 | 0 | 0 |
| ENST00000367288 | 5.24 | 1.33 | 2.17 | 3.81 | 3.06 | 4.44 | 10.25 | 7.34 | 6.09 | 4.4 |
| ENST00000424712 | 0 | 0 | 0 | 0 | 0 | 0.01 | 1.2 | 0 | 0.27 | 0.48 |
| ENST00000463049 | 0 | 0 | 0 | 0.08 | 0 | 0.2 | 0.78 | 0 | 0 | 0.3 |
| ENST00000261465 | 0 | 0 | 0 | 0 | 0 | 1.19 | 0 | 0 | 0.53 | 0.5 |
| ENST00000367027 | 2.06 | 2.02 | 0.51 | 3.15 | 3.38 | 5.94 | 7.64 | 4.05 | 3.59 | 2.33 |
| ENST00000366929 | 0.78 | 0.44 | 0.22 | 0.72 | 0.35 | 0 | 0 | 0.47 | 0 | 0 |
| ENST00000465349 | 0.64 | 0.31 | 0.34 | 0.4 | 0.41 | 0.73 | 1.57 | 0.33 | 1.53 | 1.1 |
| ENST00000272233 | 35.98 | 15.91 | 23.94 | 29.67 | 19.38 | 32.84 | 39.74 | 58.68 | 57.28 | 34.6 |
| ENST00000379066 | 3.44 | 5.3 | 7.37 | 7.34 | 6.92 | 3.44 | 3.21 | 3.59 | 3.48 | 7.38 |
| ENST00000234179 | 3.14 | 0 | 0 | 1.68 | 0.46 | 3.42 | 4.95 | 3.3 | 4.18 | 0.1 |
| ENST00000306262 | 0 | 0 | 0 | 0.03 | 0 | 0.07 | 1.02 | 0.3 | 0 | 0.5 |
| ENST00000240423 | 0.16 | 0 | 0.41 | 0.26 | 0.19 | 0 | 0 | 0 | 0 | 0 |
| ENST00000335681 | 0.18 | 0.16 | 0.08 | 0.21 | 0.07 | 1.5 | 0.92 | 0.28 | 0.14 | 0.51 |
| ENST00000474550 | 0.04 | 0.08 | 0.12 | 0.03 | 0.04 | 0.49 | 0.39 | 0.12 | 0.04 | 0.31 |
| ENST00000495559 | 0 | 0.22 | 0.03 | 0 | 0.03 | 0.45 | 0.34 | 0.1 | 0.04 | 0.36 |
| ENST00000428279 | 0.35 | 0 | 0.24 | 0 | 0.33 | 0.72 | 0.78 | 1.09 | 0.6 | 0.77 |
| ENST00000295228 | 0.35 | 0.36 | 0.51 | 0.55 | 0.45 | 1.44 | 1.6 | 0.48 | 1.08 | 0.98 |
| ENST00000452162 | 0.26 | 0.99 | 0.23 | 0.4 | 0.77 | 1.38 | 1.32 | 0.97 | 1.57 | 2.7 |
| ENST00000409184 | 0.78 | 0.46 | 1 | 0 | 0.9 | 1.66 | 0.94 | 1.14 | 1.27 | 1.05 |
| ENST00000409036 | 0 | 0 | 0 | 0 | 0 | 0.16 | 0.4 | 0 | 0.3 | 0 |
| ENST00000345739 | 3.49 | 0.5 | 2.64 | 2.85 | 2.56 | 0 | 0.55 | 1.94 | 0.77 | 1.83 |
| ENST00000417080 | 0 | 0.25 | 0.16 | 0.24 | 0 | 0.32 | 0.79 | 0.41 | 0.56 | 0.85 |
| ENST00000456655 | 0 | 0.73 | 0.43 | 1.13 | 0.63 | 2.25 | 1.33 | 1.48 | 1.72 | 1.15 |
| ENST00000412414 | 0 | 0 | 0 | 0 | 0 | 0.17 | 0.37 | 0 | 2.02 | 1.44 |
| ENST00000360351 | 0 | 0 | 0.21 | 0 | 0.21 | 0.14 | 0.41 | 0.26 | 0.41 | 0.28 |
| ENST00000419504 | 0.13 | 0.24 | 0.2 | 0.09 | 0.16 | 0.2 | 0.54 | 0.19 | 0.75 | 0.5 |
| ENST00000447924 | 8.55 | 1.57 | 0.95 | 5.12 | 4.06 | 14.84 | 18.34 | 10.02 | 13.2 | 11.76 |
| ENST00000401884 | 0 | 0 | 0 | 0 | 0 | 0 | 0.14 | 0 | 0.12 | 0.17 |
| ENST00000256509 | 0.19 | 0.32 | 0.07 | 0.44 | 0.45 | 0.84 | 0.77 | 0.92 | 0.08 | 1.45 |
| ENST00000295754 | 16.88 | 9.97 | 12.12 | 16.58 | 15.86 | 29.75 | 38.09 | 24.2 | 23.95 | 26.48 |
| ENST00000416425 | 2.95 | 1.4 | 0 | 1.86 | 1.7 | 0 | 0 | 1.23 | 0.91 | 0.28 |
| ENST00000449335 | 0 | 0 | 0 | 0 | 0 | 0.61 | 0.86 | 0 | 1.08 | 0 |
| ENST00000409697 | 0.06 | 0 | 0 | 0 | 0.74 | 0.71 | 0.2 | 1.34 | 1.1 | 0.33 |
| ENST00000358511 | 0.07 | 0 | 0.24 | 0.23 | 0 | 0 | 0 | 0.06 | 0 | 0 |
| ENST00000572787 | 0 | 1.25 | 1.49 | 2.02 | 0.13 | 0 | 0 | 0 | 0 | 0 |
| ENST00000433593 | 0.76 | 1.2 | 0.79 | 0.78 | 2.29 | 1.59 | 3.27 | 2.18 | 3.16 | 3.38 |
| ENST00000469762 | 1.24 | 0.64 | 1.73 | 0.52 | 0.92 | 0 | 0 | 0.35 | 0.53 | 0.06 |
| ENST00000464260 | 0.83 | 0.56 | 0.28 | 0.71 | 0 | 0.02 | 0.19 | 0 | 0.28 | 0.31 |
| ENST00000470834 | 0.34 | 0.37 | 0.29 | 0.46 | 0.05 | 0 | 0 | 0 | 0.16 | 0.07 |
| ENST00000538775 | 0 | 0.42 | 0.06 | 0.77 | 0 | 0.01 | 0 | 0 | 0 | 0 |
| ENST00000392692 | 0.43 | 0.02 | 0.58 | 0 | 0 | 0 | 0 | 0 | 0 | 0 |
| ENST00000486355 | 0 | 0 | 0.7 | 0 | 0.42 | 1.83 | 0.84 | 0.41 | 0.68 | 2.07 |
| ENST00000232014 | 1.99 | 0.92 | 0 | 0.49 | 0.5 | 2.49 | 4.86 | 1.89 | 1.31 | 0.49 |
| ENST00000496823 | 1.7 | 1.63 | 0.51 | 1.23 | 1.88 | 3.03 | 7.62 | 2.63 | 2.1 | 3.64 |
| ENST00000511216 | 0.85 | 0 | 0.38 | 0.26 | 0.28 | 0 | 0 | 0 | 0 | 0 |
| ENST00000399820 | 1.93 | 1.38 | 2.82 | 3.39 | 2.94 | 1.55 | 0.62 | 1.69 | 2.23 | 0.24 |
| ENST00000264888 | 0.92 | 1.18 | 3.74 | 6.99 | 0.15 | 0.34 | 0.03 | 0.84 | 0.69 | 0.16 |
| ENST00000357077 | 0.16 | 0.49 | 1.65 | 0.63 | 0.53 | 1.12 | 2.26 | 2.34 | 1.55 | 1.23 |
| ENST00000429713 | 7.08 | 4.25 | 3.79 | 7.69 | 4.19 | 8.1 | 8.88 | 12.35 | 11.12 | 10.19 |
| ENST00000504178 | 0.84 | 0.03 | 0.65 | 1.09 | 1.15 | 1.7 | 2.38 | 3.28 | 2.31 | 2.88 |
| ENST00000394439 | 6.53 | 1.97 | 4.2 | 4.99 | 4.83 | 5.22 | 9.67 | 7 | 6.82 | 9.59 |
| ENST00000437508 | 0.58 | 0.11 | 0.19 | 0 | 0 | 0 | 0 | 0 | 0.04 | 0 |
| ENST00000402744 | 8.53 | 6.95 | 4.99 | 11.14 | 11.43 | 14.27 | 17.15 | 22.28 | 26.22 | 19.48 |
| ENST00000515030 | 0 | 0 | 0 | 0 | 0 | 0.36 | 0 | 0 | 0.25 | 0 |
| ENST00000281455 | 10.9 | 18.43 | 10.68 | 11.16 | 18.72 | 40.83 | 65.38 | 12.4 | 25.68 | 20.57 |
| ENST00000284770 | 2.06 | 0.44 | 2.88 | 0.68 | 1.14 | 0 | 0.55 | 0 | 0 | 1.06 |
| ENST00000493709 | 4.64 | 0.69 | 0.68 | 1.15 | 1.22 | 0.24 | 0 | 0 | 0 | 0.11 |
| ENST00000438447 | 0 | 0 | 0 | 0 | 0 | 0.82 | 0.73 | 0 | 1.01 | 1.08 |
| ENST00000326958 | 0.52 | 1.1 | 0.63 | 0.46 | 0.74 | 2.51 | 1.42 | 1.07 | 2.46 | 1.73 |
| ENST00000505554 | 0.63 | 1.17 | 0.96 | 1.56 | 1.28 | 1.15 | 0 | 0.27 | 0 | 0.63 |
| ENST00000522633 | 0 | 0.57 | 0 | 0 | 0 | 1.07 | 2.24 | 0 | 4.21 | 0 |
| ENST00000340635 | 2.5 | 1.33 | 1.42 | 1.14 | 1.49 | 2.5 | 3.84 | 6.46 | 5.47 | 2.53 |
| ENST00000358923 | 0 | 0 | 0.19 | 0 | 0.41 | 0.49 | 0.95 | 1.25 | 0.48 | 0.2 |
| ENST00000296794 | 0.25 | 0.08 | 0.3 | 0.29 | 0 | 0.19 | 0.68 | 0.64 | 0.51 | 0.32 |
| ENST00000302351 | 1.7 | 1.67 | 0.79 | 1.65 | 1.51 | 1.8 | 2.89 | 3.51 | 3.59 | 2.66 |
| ENST00000510316 | 1.04 | 0 | 0.96 | 0.05 | 0.33 | 0 | 0 | 0 | 0 | 0 |
| ENST00000515295 | 0 | 0.46 | 0 | 0.71 | 1.48 | 2.04 | 2.63 | 0.84 | 0.61 | 1.76 |
| ENST00000509126 | 1.82 | 0.78 | 3.62 | 3.37 | 1.09 | 0 | 1.54 | 0.91 | 0.83 | 1.84 |
| ENST00000342343 | 0 | 0.08 | 0 | 0 | 0.06 | 0.19 | 0.75 | 0 | 0.07 | 0.28 |
| ENST00000515408 | 0 | 0.55 | 0.37 | 0 | 0 | 0 | 0 | 0 | 0 | 0 |
| ENST00000518189 | 0 | 0 | 0 | 0 | 0.25 | 1.32 | 3.07 | 0.23 | 0.79 | 0 |
| ENST00000519890 | 0.17 | 0.35 | 0.16 | 0.25 | 0.16 | 0.39 | 0.65 | 0.88 | 0.53 | 0.32 |
| ENST00000490094 | 2.15 | 0.3 | 1.75 | 0.23 | 1.2 | 0 | 0 | 0 | 0 | 0 |
| ENST00000448183 | 0 | 0.12 | 0.19 | 0 | 0 | 0 | 0 | 0 | 0 | 0 |
| ENST00000379284 | 0 | 0.06 | 0.61 | 0.38 | 0.23 | 0 | 0 | 0.18 | 0 | 0 |
| ENST00000379153 | 5.77 | 3.07 | 4.11 | 3.49 | 1.72 | 2.24 | 1.19 | 1.76 | 1.79 | 1.4 |
| ENST00000471353 | 0 | 1.86 | 0.47 | 1.27 | 0 | 0 | 0 | 0 | 0 | 0 |
| ENST00000377050 | 0.96 | 1.29 | 3.1 | 4.62 | 0.61 | 0.05 | 0.15 | 1.79 | 0.82 | 0.26 |
| ENST00000374975 | 272.64 | 178.75 | 18.21 | 287.03 | 25.05 | 17.29 | 7.09 | 11.43 | 111.36 | 31.34 |
| ENST00000374116 | 0 | 0.38 | 0 | 0.6 | 0 | 0 | 0 | 0 | 0 | 0 |
| ENST00000518538 | 0 | 0 | 0 | 0 | 0 | 0 | 0.78 | 1.29 | 2.3 | 0 |
| ENST00000523125 | 0 | 1.07 | 0 | 2.12 | 0 | 0 | 0 | 0 | 0 | 0 |
| ENST00000274793 | 0 | 0.49 | 2.22 | 0 | 0 | 0 | 0 | 0 | 0 | 0 |
| ENST00000449297 | 9.11 | 5.6 | 7.61 | 5.03 | 2.36 | 1.54 | 2.1 | 4.88 | 2.62 | 1.62 |
| ENST00000357489 | 0 | 0.01 | 0 | 0 | 0 | 1.68 | 1.06 | 0 | 0 | 1.22 |
| ENST00000515323 | 0.44 | 0.32 | 0 | 0.16 | 0 | 0 | 0 | 0.05 | 0 | 0 |
| ENST00000475111 | 0.78 | 0.35 | 1.46 | 0.27 | 0 | 0 | 0 | 0 | 0.44 | 0 |
| ENST00000392959 | 0 | 0 | 0 | 0 | 0 | 0 | 0.26 | 0.32 | 0 | 0 |
| ENST00000479572 | 1.02 | 0.19 | 0.81 | 0.82 | 0.02 | 0 | 0.35 | 0.18 | 0 | 0 |
| ENST00000368666 | 1.01 | 0 | 0.58 | 0 | 0 | 0 | 0 | 0 | 0 | 0 |
| ENST00000421351 | 0.5 | 0.27 | 0.56 | 0.53 | 0.78 | 0.94 | 2.7 | 1.3 | 0.5 | 0.85 |
| ENST00000297469 | 0 | 0 | 0 | 0 | 0 | 0.22 | 0.27 | 0 | 0 | 0.26 |
| ENST00000341757 | 0.21 | 0.27 | 0 | 0.11 | 0 | 0 | 0 | 0 | 0 | 0 |
| ENST00000496855 | 1.86 | 0.34 | 1.77 | 2.11 | 1.3 | 0 | 0 | 0 | 1.06 | 0.71 |
| ENST00000396376 | 5.29 | 7.19 | 4.32 | 9.55 | 5.41 | 3.42 | 1.23 | 3.09 | 8.46 | 0.8 |
| ENST00000419661 | 0 | 0 | 0 | 0 | 0 | 0 | 1.19 | 1.38 | 1.99 | 0 |
| ENST00000476120 | 0.25 | 0.02 | 0.41 | 0 | 0.1 | 0 | 0 | 0 | 0 | 0 |
| ENST00000444627 | 1.85 | 0 | 0 | 0 | 0.35 | 1.38 | 1.54 | 1.8 | 0.24 | 2.47 |
| ENST00000005178 | 9.53 | 7.18 | 5.49 | 11.57 | 29.25 | 27.7 | 48.88 | 23.41 | 34.9 | 9.83 |
| ENST00000468445 | 0.92 | 0.2 | 0.19 | 0.66 | 1.34 | 1.54 | 5.78 | 1.36 | 4 | 0.8 |
| ENST00000341441 | 1.02 | 0.87 | 0 | 0 | 0 | 0 | 0 | 0 | 0 | 0 |
| ENST00000463263 | 0 | 0 | 0 | 1.09 | 0.21 | 1.55 | 2.37 | 1.53 | 2.36 | 1.69 |
| ENST00000446198 | 0.95 | 0.88 | 0.96 | 0 | 0.45 | 0.46 | 3.26 | 2.12 | 1.67 | 2.52 |
| ENST00000321063 | 0.34 | 0.68 | 0.13 | 0.51 | 0.72 | 0.94 | 1.37 | 0.6 | 1.54 | 0.69 |
| ENST00000359827 | 0.43 | 0.34 | 0.18 | 0 | 0 | 0.82 | 0.65 | 0.5 | 0.54 | 0.98 |
| ENST00000423507 | 0.47 | 0.66 | 0.2 | 0.39 | 0.28 | 1.29 | 0.64 | 1.19 | 1.11 | 1.06 |
| ENST00000422328 | 0.59 | 0 | 0.47 | 0.4 | 0 | 0 | 0 | 0 | 0 | 0 |
| ENST00000223271 | 5.18 | 4.23 | 10.78 | 6.07 | 6.29 | 21.69 | 18.52 | 5.68 | 12.93 | 10.42 |
| ENST00000276326 | 0.4 | 0.41 | 0.32 | 2.28 | 0.71 | 1.72 | 2.97 | 2.42 | 0.8 | 2.38 |
| ENST00000521516 | 0 | 0 | 0 | 0 | 0 | 0.36 | 0.41 | 0 | 0 | 0.39 |
| ENST00000220772 | 30.3 | 10.79 | 12.06 | 13.38 | 12.3 | 25.47 | 42.12 | 25.5 | 33.58 | 36.76 |
| ENST00000379845 | 6.53 | 8.33 | 6.46 | 13.74 | 10.04 | 15.23 | 21.9 | 13.33 | 15.83 | 23.05 |
| ENST00000352041 | 0 | 0 | 0 | 0 | 0 | 1 | 0 | 0 | 0.65 | 0.05 |
| ENST00000521661 | 0 | 0 | 0.84 | 0 | 0.35 | 0 | 0 | 0 | 0 | 0 |
| ENST00000337919 | 0 | 0 | 0 | 0 | 0 | 0 | 0.37 | 0 | 0.37 | 0.37 |
| ENST00000256104 | 5.74 | 15.51 | 10.11 | 7.61 | 42.27 | 100.08 | 112.86 | 12.56 | 40.83 | 34.44 |
| ENST00000276609 | 0.07 | 0 | 0 | 0 | 0 | 0.05 | 0.11 | 0.09 | 0.14 | 0.14 |
| ENST00000532255 | 0.06 | 0.12 | 0.52 | 0.2 | 0.23 | 0.15 | 0 | 0 | 0 | 0 |
| ENST00000381434 | 2.3 | 4.41 | 5.19 | 7.33 | 7.51 | 10.32 | 10.47 | 8.11 | 8.63 | 6.2 |
| ENST00000380875 | 0 | 0.07 | 0 | 0.15 | 0.02 | 0.54 | 0.67 | 0.02 | 0 | 0.47 |
| ENST00000259605 | 0 | 0 | 0 | 0 | 0 | 0.43 | 3.99 | 0 | 0 | 0 |
| ENST00000395882 | 2.98 | 1.68 | 0.6 | 2.92 | 1.01 | 5.03 | 8.28 | 5.61 | 3.15 | 2.63 |
| ENST00000359847 | 2.37 | 1.37 | 0.55 | 2.72 | 5.31 | 3.53 | 8.2 | 3.5 | 4.41 | 5.5 |
| ENST00000394353 | 0 | 0 | 1.49 | 0.83 | 0 | 0 | 0 | 0 | 0 | 0 |
| ENST00000477104 | 0 | 1.26 | 1.24 | 0 | 0 | 0 | 0 | 0 | 0 | 0 |
| ENST00000373755 | 0 | 0 | 0.78 | 0.77 | 0 | 0 | 0 | 0 | 0 | 0 |
| ENST00000373754 | 0.87 | 0 | 0 | 0 | 0.39 | 0.63 | 1.37 | 0.69 | 0.68 | 2.25 |
| ENST00000373276 | 0 | 0 | 0 | 0.43 | 0.25 | 0.55 | 0.25 | 0.61 | 0.78 | 0.54 |
| ENST00000463535 | 2.09 | 0.1 | 2.09 | 1.02 | 0.96 | 2.69 | 3.41 | 1.92 | 3.69 | 3.71 |
| ENST00000459858 | 0 | 0 | 0 | 0 | 0 | 0.56 | 1.33 | 0.44 | 0.27 | 0.58 |
| ENST00000356818 | 0 | 0 | 0 | 0 | 0 | 0 | 0.78 | 0 | 0 | 0.45 |
| ENST00000401470 | 0.48 | 0.21 | 0.42 | 0 | 0.41 | 0 | 0 | 0 | 0.19 | 0 |
| ENST00000263056 | 0 | 0 | 0.06 | 0 | 0 | 1.09 | 1.83 | 0 | 1.01 | 0 |
| ENST00000542815 | 3.33 | 0.5 | 0 | 0 | 0 | 0 | 0 | 0 | 0 | 0 |
| ENST00000298295 | 9.46 | 6.41 | 9.82 | 19.46 | 15.45 | 31.11 | 27.16 | 18.29 | 31.34 | 15.48 |
| ENST00000424265 | 0 | 0 | 0 | 0 | 0 | 0 | 0.21 | 0 | 0.58 | 0.11 |
| ENST00000348795 | 0.15 | 0 | 0 | 0 | 1.33 | 0.61 | 3.54 | 1.01 | 1.66 | 0.76 |
| ENST00000371837 | 0 | 0 | 0 | 0 | 0 | 0.6 | 1.69 | 0 | 0.79 | 0 |
| ENST00000428800 | 0.65 | 0.65 | 1.58 | 0.79 | 1.48 | 0.35 | 0 | 0.86 | 0 | 0 |
| ENST00000356016 | 0 | 0 | 0 | 0 | 0 | 0 | 3.05 | 0 | 0 | 1.36 |
| ENST00000369252 | 1.49 | 0.64 | 2.07 | 2.14 | 1.51 | 2.91 | 5.23 | 3.34 | 2.25 | 1.96 |
| ENST00000493766 | 0 | 0 | 0.92 | 1.54 | 0.35 | 0 | 0 | 0 | 0 | 0 |
| ENST00000515603 | 0 | 0 | 0 | 0 | 0 | 0 | 0.88 | 0 | 0 | 0.33 |
| ENST00000408968 | 49.83 | 53.15 | 33.3 | 61.91 | 52.43 | 66.34 | 90.21 | 82.93 | 88.51 | 75.79 |
| ENST00000347936 | 0.43 | 0.34 | 0.75 | 0.56 | 0.24 | 0 | 0 | 0 | 0 | 0 |
| ENST00000312221 | 0.12 | 0.7 | 0 | 0.03 | 0 | 0.85 | 1.09 | 0.94 | 1.96 | 0.63 |
| ENST00000403482 | 0 | 0.8 | 0.32 | 0.66 | 0 | 1.98 | 3.52 | 0.62 | 0.55 | 1.06 |
| ENST00000529986 | 0.6 | 0.72 | 1.62 | 1.38 | 1.22 | 1.98 | 2.57 | 2.24 | 2.11 | 1.63 |
| ENST00000521849 | 1.35 | 0.39 | 1.94 | 0.41 | 0 | 0 | 0.46 | 0 | 0.12 | 0 |
| ENST00000530124 | 0 | 0 | 0.38 | 0 | 0.71 | 1.61 | 1.66 | 1.1 | 0.75 | 0.7 |
| ENST00000528487 | 2.26 | 1.16 | 1.44 | 1.56 | 0.94 | 0.04 | 0.55 | 0 | 0 | 1.62 |
| ENST00000540843 | 2.17 | 0 | 1.57 | 1.13 | 0 | 0 | 0 | 0 | 0 | 0 |
| ENST00000531380 | 9.25 | 5.66 | 8.39 | 12.27 | 10.01 | 14.04 | 25.81 | 13.12 | 18.92 | 12.53 |
| ENST00000531452 | 0.02 | 0 | 0.13 | 0 | 0 | 1.4 | 1.93 | 0 | 0.31 | 2.22 |
| ENST00000264036 | 15.23 | 6.17 | 12.17 | 9.33 | 6.18 | 17.97 | 23.11 | 11.47 | 11.66 | 30.32 |
| ENST00000545331 | 0.34 | 0 | 0 | 0 | 0 | 0.18 | 0.87 | 0.23 | 0.92 | 0.8 |
| ENST00000541453 | 0 | 3.52 | 0 | 2.24 | 0 | 0 | 0 | 0 | 0 | 0 |
| ENST00000447609 | 0.54 | 0 | 0.34 | 0.02 | 0 | 0.22 | 0.82 | 0.95 | 0.67 | 0.52 |
| ENST00000451599 | 0.43 | 0.19 | 0.18 | 0.35 | 0 | 0 | 0 | 0.13 | 0 | 0 |
| ENST00000550386 | 0 | 0 | 0 | 0 | 0 | 0 | 0.37 | 0.42 | 0.64 | 0.45 |
| ENST00000551301 | 0.55 | 0.36 | 1.01 | 0 | 0 | 0 | 0 | 0 | 0 | 0 |
| ENST00000552189 | 0.21 | 0.57 | 0 | 0 | 0.23 | 0.9 | 0.78 | 0.24 | 0.7 | 0.38 |
| ENST00000257895 | 0.57 | 0 | 0 | 0 | 0 | 3.47 | 2.85 | 0 | 1.13 | 0 |
| ENST00000550770 | 0 | 0.54 | 0.84 | 0.33 | 0.41 | 0 | 0 | 0 | 0 | 0 |
| ENST00000261187 | 3.23 | 2.81 | 3.4 | 5.12 | 4.61 | 6.93 | 10.48 | 4.76 | 5.14 | 5.44 |
| ENST00000258526 | 9.68 | 5.14 | 10 | 9.31 | 4.69 | 2.48 | 3.99 | 5.15 | 7.03 | 8.36 |
| ENST00000553059 | 0.55 | 0.35 | 0 | 0.25 | 0 | 0 | 0 | 0 | 0 | 0 |
| ENST00000312777 | 0 | 0 | 0.34 | 0.25 | 0 | 0 | 0 | 0 | 0 | 0 |
| ENST00000542939 | 10.73 | 0 | 6.16 | 0 | 0 | 0 | 0.66 | 0 | 0 | 0 |
| ENST00000543318 | 0 | 0 | 0 | 0 | 0 | 1.12 | 0.31 | 0 | 0.89 | 0.22 |
| ENST00000542067 | 2.62 | 4.56 | 2.62 | 3.39 | 4.52 | 0 | 0 | 0 | 0 | 0 |
| ENST00000310336 | 0.33 | 0.22 | 0 | 0 | 0 | 0 | 0 | 0 | 0 | 0 |
| ENST00000397858 | 0 | 0 | 0 | 0 | 0 | 1.06 | 1.56 | 0 | 0 | 0 |
| ENST00000553002 | 0.61 | 0.56 | 0.31 | 0 | 0 | 0 | 0 | 0 | 0 | 0 |
| ENST00000396618 | 0.56 | 0.29 | 0 | 0.87 | 0 | 1.46 | 5.06 | 1.3 | 1.71 | 0 |
| ENST00000556281 | 0 | 0 | 0 | 0.8 | 1.02 | 3.13 | 1.52 | 2.12 | 0 | 1.34 |
| ENST00000554093 | 2.47 | 1.62 | 1.41 | 1.29 | 1.81 | 0 | 0 | 0 | 1.08 | 0 |
| ENST00000409949 | 0 | 0 | 0 | 0 | 0 | 0.73 | 0.19 | 0 | 0 | 0.75 |
| ENST00000556154 | 1.48 | 0.97 | 0.59 | 2.05 | 2 | 5.03 | 3.9 | 2.04 | 1.72 | 3.08 |
| ENST00000557609 | 0 | 0 | 0 | 0 | 0 | 0 | 0.4 | 0 | 0.5 | 0 |
| ENST00000556045 | 0 | 0 | 0.24 | 0.15 | 0.17 | 0 | 0 | 0 | 0 | 0 |
| ENST00000329146 | 0 | 0 | 0 | 0 | 0 | 0 | 0.59 | 0.48 | 0 | 0 |
| ENST00000550577 | 9.29 | 2.85 | 11.88 | 6.09 | 6.07 | 6.99 | 21.01 | 13.34 | 8.96 | 17.44 |
| ENST00000538259 | 0 | 0 | 0 | 0 | 0 | 0 | 1.58 | 0 | 1.94 | 0 |
| ENST00000566718 | 2.77 | 0.27 | 1.54 | 0.17 | 0.74 | 0 | 0.26 | 0 | 0 | 0.53 |
| ENST00000316623 | 49.28 | 36.23 | 62.43 | 64.02 | 50.58 | 88.01 | 121.39 | 62.8 | 49.7 | 102.08 |
| ENST00000399228 | 0 | 0 | 1.15 | 1.2 | 0.57 | 0 | 0 | 0 | 0 | 0.09 |
| ENST00000560121 | 0 | 0 | 0 | 0.21 | 0 | 0.15 | 0.93 | 0.57 | 1.32 | 0.37 |
| ENST00000559397 | 0.55 | 2.31 | 2.6 | 0.29 | 5.05 | 0 | 0 | 0 | 0.19 | 0 |
| ENST00000404484 | 0 | 1.35 | 1.66 | 0 | 0.07 | 0 | 0 | 0 | 0 | 0 |
| ENST00000319212 | 0 | 1.23 | 0 | 0 | 0 | 1.67 | 1.78 | 0.2 | 0.73 | 1.27 |
| ENST00000560523 | 0 | 0 | 0 | 0 | 0 | 0 | 0.99 | 0 | 0 | 1.15 |
| ENST00000389093 | 1.78 | 0.75 | 0 | 0 | 0 | 3.18 | 1.75 | 0 | 7.73 | 2.52 |
| ENST00000395135 | 0.32 | 0.44 | 0.65 | 0 | 0 | 0 | 0 | 0 | 0 | 0 |
| ENST00000268150 | 7.68 | 2.91 | 14.09 | 4.37 | 7.21 | 11.1 | 23.45 | 19.75 | 7.62 | 17.62 |
| ENST00000560738 | 0 | 0 | 0.18 | 0 | 1.11 | 0.65 | 1.37 | 0 | 1.47 | 1.56 |
| ENST00000262376 | 0.35 | 0.52 | 0.54 | 0.27 | 0.31 | 1.01 | 1.92 | 0.67 | 0.31 | 1.78 |
| ENST00000563641 | 0 | 0.09 | 0 | 0 | 0 | 0 | 0.46 | 0 | 1.09 | 0.93 |
| ENST00000334350 | 1.53 | 0.53 | 0.02 | 0.35 | 1.55 | 2.73 | 2.36 | 1.86 | 5.61 | 0.68 |
| ENST00000568327 | 0.61 | 0.29 | 0.41 | 0.17 | 0.3 | 0 | 0.07 | 0 | 0 | 0 |
| ENST00000563432 | 2.03 | 3.69 | 1.97 | 3.63 | 1.4 | 0.71 | 0.72 | 0.05 | 1.64 | 0 |
| ENST00000568079 | 0.31 | 0.42 | 0.63 | 0 | 0.5 | 0 | 0 | 0.14 | 0.03 | 0 |
| ENST00000572293 | 0 | 0 | 0 | 0 | 0 | 0 | 0.76 | 0 | 0.75 | 0 |
| ENST00000397317 | 0 | 0 | 0 | 0 | 0.14 | 0.32 | 0.89 | 0 | 1.01 | 0.83 |
| ENST00000336708 | 1.04 | 0.96 | 2.02 | 0.94 | 0.66 | 0.67 | 0.8 | 0.12 | 0.01 | 0.05 |
| ENST00000225688 | 5 | 1.9 | 1.09 | 1.32 | 2.4 | 9.78 | 8.67 | 6.44 | 5.34 | 6.46 |
| ENST00000531253 | 0 | 0.79 | 0.08 | 0 | 0.19 | 0 | 0 | 0 | 0 | 0 |
| ENST00000269593 | 217.25 | 175.83 | 95.26 | 161.29 | 131.72 | 287.69 | 314.64 | 212.43 | 294.1 | 346.15 |
| ENST00000571864 | 2.17 | 1.15 | 1.34 | 1.31 | 0.16 | 0 | 0 | 0 | 0 | 0 |
| ENST00000506582 | 1.5 | 0.85 | 0.25 | 0.82 | 0 | 0 | 0 | 0 | 0 | 0 |
| ENST00000477179 | 0 | 0 | 0 | 0 | 0 | 2 | 1.12 | 0.82 | 0.64 | 0.76 |
| ENST00000290863 | 0 | 0 | 0.47 | 0.08 | 0 | 0.36 | 0.4 | 0.56 | 1 | 0 |
| ENST00000335221 | 0.99 | 0.2 | 0 | 0 | 0 | 0 | 0 | 0 | 0 | 0 |
| ENST00000585981 | 0 | 0 | 0 | 2.01 | 0.94 | 6.64 | 5.86 | 3.94 | 4.33 | 3.27 |
| ENST00000428549 | 0.97 | 0.8 | 0 | 1.46 | 0.72 | 1.86 | 4.33 | 2.49 | 1.68 | 1.18 |
| ENST00000335793 | 3.14 | 0.84 | 1.4 | 4.49 | 2.7 | 4.97 | 4.41 | 4.72 | 4.7 | 3.77 |
| ENST00000545227 | 0.15 | 0 | 0.14 | 0.18 | 0 | 0 | 0 | 0 | 0 | 0 |
| ENST00000579662 | 0 | 0 | 0 | 0 | 0 | 0.1 | 0 | 0 | 0.22 | 0 |
| ENST00000200181 | 0 | 0 | 0 | 0 | 0 | 0.11 | 0.21 | 0.16 | 0 | 0 |
| ENST00000450894 | 0 | 0 | 0 | 0 | 0 | 0 | 1.31 | 0.98 | 0 | 0 |
| ENST00000587127 | 0.68 | 0.51 | 0.23 | 0.98 | 0.64 | 0 | 0.01 | 0.42 | 0 | 0.71 |
| ENST00000588087 | 0.77 | 0 | 0.65 | 0.56 | 0.17 | 0 | 0.17 | 0.1 | 0.05 | 0 |
| ENST00000591914 | 0 | 0 | 0 | 0 | 0 | 0.95 | 0 | 0.29 | 0.5 | 0.16 |
| ENST00000301286 | 8.74 | 11.13 | 6.1 | 5.95 | 24.37 | 55.97 | 94.61 | 6.39 | 40.71 | 27.16 |
| ENST00000344979 | 0 | 0 | 0 | 0 | 0 | 0 | 0.25 | 0 | 0.34 | 0.13 |
| ENST00000595068 | 0.53 | 0.4 | 0.54 | 1.07 | 0.93 | 0 | 0 | 0.39 | 0.54 | 0 |
| ENST00000540605 | 0.37 | 0 | 0.69 | 0 | 0.28 | 0 | 0 | 0 | 0 | 0 |
| ENST00000348817 | 0 | 0 | 0 | 0 | 0 | 0.59 | 1.25 | 0 | 0 | 0.93 |
| ENST00000409587 | 0.77 | 0.35 | 0.73 | 0 | 1.6 | 0.04 | 0 | 0 | 0.08 | 0.45 |
| ENST00000598055 | 1.75 | 0.66 | 0.66 | 1.49 | 0 | 0 | 0 | 0 | 0 | 0 |
| ENST00000588742 | 0 | 0.95 | 1.11 | 2.06 | 0 | 0 | 0 | 0 | 0 | 0 |
| ENST00000291270 | 3.59 | 1.4 | 1.86 | 1.74 | 1.1 | 0.59 | 1.14 | 1.29 | 1.24 | 1.16 |
| ENST00000601519 | 0 | 0 | 0 | 0 | 0 | 0.94 | 1.77 | 0.19 | 0 | 1.17 |
| ENST00000543227 | 1.4 | 0.48 | 0 | 1.62 | 0 | 1.33 | 1.53 | 1.39 | 1.02 | 2.49 |
| ENST00000540744 | 0.72 | 0.51 | 0.82 | 0 | 1.23 | 0 | 0 | 0 | 0 | 0 |
| ENST00000377813 | 4.97 | 4.15 | 4.77 | 3.13 | 3.89 | 6.97 | 7.78 | 8.61 | 7.25 | 10.39 |
| ENST00000420488 | 0 | 0 | 0 | 0 | 0 | 0 | 0.98 | 0 | 0.8 | 0.82 |
| ENST00000342427 | 0 | 0 | 0 | 0 | 0 | 0 | 0.17 | 0.16 | 0.14 | 0 |
| ENST00000447406 | 0.32 | 0.51 | 0.5 | 0 | 0 | 0 | 0 | 0 | 0 | 0 |
| ENST00000217407 | 5.85 | 4.1 | 0.15 | 2.09 | 5.14 | 18.72 | 6.03 | 5.65 | 8.26 | 14.56 |
| ENST00000338380 | 4.99 | 11.05 | 0.71 | 5.9 | 17.32 | 28.47 | 43.82 | 15.53 | 26.34 | 12.23 |
| ENST00000327979 | 0.06 | 0.1 | 0.08 | 0.28 | 0.25 | 1.14 | 0.69 | 0.49 | 0.74 | 0.71 |
| ENST00000398960 | 0 | 0 | 0 | 0 | 0 | 1.21 | 0 | 0 | 0.42 | 0 |
| ENST00000398646 | 0 | 0 | 0 | 0 | 0 | 0.91 | 0.46 | 0 | 0 | 1.63 |
| ENST00000352133 | 0.52 | 0.67 | 0.18 | 0.49 | 0.21 | 0.12 | 0.15 | 0.12 | 0.3 | 0 |
| ENST00000479117 | 0 | 0 | 0 | 0 | 0.14 | 0.63 | 0.76 | 0.32 | 0.73 | 0 |
| ENST00000523323 | 0 | 1.05 | 0.5 | 0 | 0.51 | 0 | 0 | 0 | 0 | 0 |
| ENST00000334538 | 0.12 | 0 | 0 | 0.01 | 0 | 0.15 | 0.2 | 0.14 | 0.92 | 0.11 |
| ENST00000337612 | 0 | 0 | 0 | 0 | 0.2 | 0.42 | 0.75 | 0.77 | 0 | 0.34 |
| ENST00000461809 | 0 | 0 | 0 | 0 | 0 | 0.15 | 0.14 | 0.12 | 0.26 | 0 |
| ENST00000344710 | 0 | 0 | 0 | 0 | 0 | 0.21 | 0.11 | 0 | 0.17 | 0.03 |
| ENST00000407965 | 0.71 | 0 | 0.63 | 0 | 0 | 0 | 0 | 0 | 0 | 0 |
| ENST00000440761 | 0 | 0 | 0 | 0 | 0 | 0.35 | 0 | 0 | 0.67 | 0 |
| ENST00000407236 | 0.89 | 1.37 | 0.89 | 0 | 1.24 | 0 | 0 | 0 | 0.53 | 0 |
| ENST00000329363 | 0 | 0 | 0 | 0 | 0 | 0.92 | 1.31 | 0 | 0.42 | 0 |
| ENST00000312108 | 0 | 0 | 0.48 | 0.06 | 0 | 0.81 | 0.59 | 1.27 | 1.29 | 0.98 |
| ENST00000217964 | 0.28 | 0 | 0 | 0 | 0.25 | 0.58 | 0.43 | 0 | 0.9 | 1.56 |
| ENST00000463236 | 0 | 0 | 0 | 0 | 0 | 0 | 1.75 | 0 | 0.29 | 0.78 |
| ENST00000441703 | 0 | 0.26 | 0.45 | 0 | 0.43 | 1.08 | 2.47 | 2.49 | 0 | 1.5 |
| ENST00000343533 | 0 | 0.35 | 0.71 | 0 | 0.56 | 0 | 0 | 0.27 | 0 | 0 |
| ENST00000372384 | 0 | 0 | 0 | 0 | 0 | 0.38 | 0 | 0 | 0.6 | 0 |
| ENST00000370674 | 0 | 0.39 | 0 | 0.64 | 2.11 | 8.07 | 5.1 | 1.02 | 2.95 | 1.89 |
| ENST00000370477 | 0 | 0.73 | 1.31 | 1.62 | 1.81 | 0.43 | 0 | 0.68 | 0 | 0.53 |
|  |  |  |  |  |  |  |  |  |  |  |
