# Supplementary material for: Prediction of MicroRNA and Gene Target in Synovium-Associated Pain of Knee Osteoarthritis Based on Canonical Correlation Analysis
Source: Biomed Res Int. 2019 Oct 13;2019:4506876. doi: 10.1155/2019/4506876 (PMC6815580; doi:10.1155/2019/4506876)
Supplement: Supplementary Materials — Appendix 1: four matrixes. We selected the correlation coefficients of the original variable and the first canonical variable because the first canonical variable had the highest canonical correlation coefficient of 0.954. Appendixes 2 and 3: the miRNAs and mRNAs selected in this study, which will be further used in network construction. Appendix 4: 13 miRNAs and 26 mRNAs identified by CCA, which were analyzed by the Spearman rank correlation test. [file 4506876.f1.zip › 4506876.f1/appendix 4_BMRI_2859738.docx]

| Appendix 4 | | | | | | | |
| --- | --- | --- | --- | --- | --- | --- | --- |
| miRNA | GeneName | correlation | pvalue | miRNA | GeneName | correlation | pvalue |
| hsa-miR-133a-3p | MIB2 | 0.665903 | 0.035552 | hsa-miR-133a-3p | RNF38 | 0.700649 | 0.024019 |
| hsa-miR-145-5p | MIB2 | 0.769345 | 0.009274 | hsa-miR-215-5p | RNF38 | 0.700649 | 0.024019 |
| hsa-miR-133a-3p | THEMIS2 | 0.762873 | 0.010272 | hsa-miR-224-5p | RNF38 | 0.700649 | 0.024019 |
| hsa-miR-335-5p | THEMIS2 | 0.787885 | 0.006795 | hsa-miR-145-5p | RNF38 | 0.700649 | 0.024019 |
| hsa-miR-133a-3p | LCK | -0.65548 | 0.039633 | hsa-miR-133a-3p | NTRK2 | 0.842424 | 0.004459 |
| hsa-miR-224-5p | LCK | -0.64182 | 0.04544 | hsa-miR-145-5p | NTRK2 | 0.781818 | 0.011651 |
| hsa-miR-145-5p | LCK | -0.68279 | 0.029563 | hsa-miR-145-5p | NTRK2 | 0.648485 | 0.049043 |
| hsa-miR-335-5p | LCK | -0.72376 | 0.017965 | hsa-miR-335-5p | NTRK2 | 0.842424 | 0.004459 |
| hsa-miR-224-5p | MACF1 | -0.78163 | 0.007571 | hsa-miR-145-5p | GSN | -0.68335 | 0.029377 |
| hsa-miR-215-5p | MACF1 | -0.68158 | 0.029966 | hsa-miR-133a-3p | GSN | -0.70065 | 0.024019 |
| hsa-miR-133a-3p | TMEM53 | 0.860314 | 0.001402 | hsa-miR-335-5p | GSN | -0.68335 | 0.029377 |
| hsa-miR-224-5p | TMEM53 | 0.669133 | 0.034348 | hsa-miR-224-5p | UCK1 | 0.652973 | 0.040658 |
| hsa-miR-145-5p | TMEM53 | 0.737412 | 0.014937 | hsa-miR-224-5p | MAP3K8 | 0.634994 | 0.048546 |
| hsa-miR-224-5p | TMEM53 | -0.64406 | 0.04445 | hsa-miR-215-5p | MAP3K8 | 0.696445 | 0.025253 |
| hsa-miR-145-5p | LEPR | 0.64438 | 0.044312 | hsa-miR-133a-3p | C10orf10 | 0.733333 | 0.021166 |
| hsa-miR-133a-3p | LEPR | 0.814593 | 0.004106 | hsa-miR-335-5p | C10orf10 | 0.781818 | 0.011651 |
| hsa-miR-335-5p | LEPR | 0.826752 | 0.00318 | hsa-miR-145-5p | SNCG | 0.730116 | 0.016506 |
| hsa-miR-145-5p | LEPR | 0.781818 | 0.011651 | hsa-miR-133a-3p | SNCG | 0.754658 | 0.011645 |
| hsa-miR-133a-3p | LEPR | 0.793939 | 0.009844 | hsa-miR-335-5p | SNCG | 0.803741 | 0.005084 |
| hsa-miR-335-5p | POU2F1 | 0.64438 | 0.044312 | hsa-miR-133a-3p | LIPA | 0.723297 | 0.018074 |
| hsa-miR-133a-3p | LMOD1 | 0.830303 | 0.005557 | hsa-miR-335-5p | LIPA | 0.753123 | 0.011915 |
| hsa-miR-145-5p | LMOD1 | 0.757576 | 0.015921 | hsa-miR-215-5p | LIPA | 0.797863 | 0.005677 |
| hsa-miR-224-5p | PIK3C2B | 0.696445 | 0.025253 | hsa-miR-224-5p | LIPA | 0.723297 | 0.018074 |
| hsa-miR-335-5p | PIK3C2B | 0.648649 | 0.04247 | hsa-miR-224-5p | LIPA | -0.63392 | 0.049046 |
| hsa-miR-145-5p | HSD11B1 | 0.890909 | 0.00138 | hsa-miR-133a-3p | ABLIM1 | 0.793939 | 0.009844 |
| hsa-miR-133a-3p | HSD11B1 | 0.951515 | 0 | hsa-miR-133a-3p | IFITM1 | 0.648485 | 0.049043 |
| hsa-miR-335-5p | HSD11B1 | 0.818182 | 0.006811 | hsa-miR-335-5p | IFITM1 | 0.684848 | 0.035092 |
| hsa-miR-224-5p | TGFB2 | -0.75662 | 0.011306 | hsa-miR-133a-3p | SLC22A18 | -0.67883 | 0.030899 |
| hsa-miR-215-5p | LYST | 0.660606 | 0.044027 | hsa-miR-224-5p | SLC22A18 | -0.69176 | 0.026678 |
| hsa-miR-335-5p | LYST | 0.757576 | 0.015921 | hsa-miR-145-5p | SLC22A18 | -0.69176 | 0.026678 |
| hsa-miR-133a-3p | RHOB | 0.648485 | 0.049043 | hsa-miR-224-5p | SLC22A18 | 0.650459 | 0.041705 |
| hsa-miR-145-5p | PRKD3 | -0.74164 | 0.014075 | hsa-miR-224-5p | ARNTL | 0.680854 | 0.030211 |
| hsa-miR-133a-3p | PRKD3 | -0.74772 | 0.0129 | hsa-miR-133a-3p | CTNND1 | 0.69697 | 0.031141 |
| hsa-miR-215-5p | PRKD3 | -0.6383 | 0.047024 | hsa-miR-145-5p | AHNAK | 0.803741 | 0.005084 |
| hsa-miR-145-5p | PRKD3 | 0.802435 | 0.005211 | hsa-miR-133a-3p | AHNAK | 0.865096 | 0.001227 |
| hsa-miR-133a-3p | PRKD3 | 0.924016 | 0.000133 | hsa-miR-335-5p | AHNAK | 0.668762 | 0.034485 |
| hsa-miR-335-5p | PRKD3 | 0.753803 | 0.011795 | hsa-miR-224-5p | AHNAK | 0.656491 | 0.039223 |
| hsa-miR-224-5p | NCAPH | -0.7579 | 0.011089 | hsa-miR-133a-3p | SYVN1 | -0.65654 | 0.039204 |
| hsa-miR-224-5p | NPAS2 | 0.664694 | 0.03601 | hsa-miR-224-5p | NUMA1 | -0.76804 | 0.00947 |
| hsa-miR-224-5p | NPAS2 | 0.841479 | 0.002271 | hsa-miR-133a-3p | FZD4 | 0.854545 | 0.003505 |
| hsa-miR-145-5p | IL1R1 | 0.741645 | 0.014075 | hsa-miR-145-5p | FZD4 | 0.721212 | 0.024195 |
| hsa-miR-133a-3p | IL1R1 | 0.717329 | 0.019528 | hsa-miR-335-5p | FZD4 | 0.818182 | 0.006811 |
| hsa-miR-335-5p | INHBB | 0.672727 | 0.039381 | hsa-miR-224-5p | ITPR2 | -0.69176 | 0.026678 |
| hsa-miR-224-5p | PKP4 | 0.733333 | 0.021166 | hsa-miR-335-5p | ITPR2 | -0.70469 | 0.02287 |
| hsa-miR-215-5p | RAPGEF4 | 0.797863 | 0.005677 | hsa-miR-335-5p | SLC48A1 | -0.79786 | 0.005677 |
| hsa-miR-133a-3p | RAPGEF4 | 0.723297 | 0.018074 | hsa-miR-215-5p | SENP1 | 0.765961 | 0.009787 |
| hsa-miR-224-5p | RAPGEF4 | 0.723297 | 0.018074 | hsa-miR-224-5p | SENP1 | 0.942254 | 4.54E-05 |
| hsa-miR-335-5p | RAPGEF4 | 0.753123 | 0.011915 | hsa-miR-145-5p | RDH5 | 0.641821 | 0.04544 |
| hsa-miR-224-5p | ATF2 | -0.93939 | 0 | hsa-miR-215-5p | RDH5 | 0.778379 | 0.007998 |
| hsa-miR-133a-3p | ATF2 | 0.648485 | 0.049043 | hsa-miR-133a-3p | RDH5 | 0.716928 | 0.019629 |
| hsa-miR-224-5p | TNS1 | 0.77204 | 0.00888 | hsa-miR-145-5p | PRIM1 | -0.68962 | 0.02735 |
| hsa-miR-133a-3p | ACKR3 | 0.806061 | 0.008236 | hsa-miR-133a-3p | PRIM1 | -0.68962 | 0.02735 |
| hsa-miR-335-5p | ACKR3 | 0.721212 | 0.024195 | hsa-miR-133a-3p | SLC16A7 | 0.684848 | 0.035092 |
| hsa-miR-145-5p | ACKR3 | 0.757576 | 0.015921 | hsa-miR-335-5p | SLC16A7 | 0.757576 | 0.015921 |
| hsa-miR-133a-3p | TGFBR2 | 0.781818 | 0.011651 | hsa-miR-145-5p | PLXNC1 | -0.70909 | 0.027514 |
| hsa-miR-145-5p | TGFBR2 | 0.769697 | 0.013672 | hsa-miR-224-5p | PLXNC1 | -0.80606 | 0.008236 |
| hsa-miR-133a-3p | BBX | 0.678557 | 0.030994 | hsa-miR-215-5p | PLXNC1 | -0.68485 | 0.035092 |
| hsa-miR-224-5p | BBX | 0.678557 | 0.030994 | hsa-miR-215-5p | P2RX4 | 0.703272 | 0.02327 |
| hsa-miR-215-5p | BBX | 0.768037 | 0.00947 | hsa-miR-224-5p | P2RX4 | 0.723756 | 0.017965 |
| hsa-miR-335-5p | BBX | 0.73821 | 0.014771 | hsa-miR-335-5p | P2RX4 | 0.648649 | 0.04247 |
| hsa-miR-145-5p | CCDC14 | 0.631949 | 0.049975 | hsa-miR-133a-3p | P2RX4 | -0.64875 | 0.042428 |
| hsa-miR-133a-3p | CCDC14 | 0.656491 | 0.039223 | hsa-miR-145-5p | NDRG2 | 0.700649 | 0.024019 |
| hsa-miR-224-5p | COL6A6 | -0.84666 | 0.002002 | hsa-miR-133a-3p | NDRG2 | 0.700649 | 0.024019 |
| hsa-miR-215-5p | COL6A6 | -0.73058 | 0.016402 | hsa-miR-215-5p | NDRG2 | 0.700649 | 0.024019 |
| hsa-miR-133a-3p | TOPBP1 | -0.64865 | 0.04247 | hsa-miR-224-5p | NDRG2 | 0.700649 | 0.024019 |
| hsa-miR-145-5p | TOPBP1 | -0.70327 | 0.02327 | hsa-miR-335-5p | LRP10 | -0.75312 | 0.011915 |
| hsa-miR-133a-3p | TNIK | -0.79147 | 0.006377 | hsa-miR-133a-3p | COCH | 0.7792 | 0.007889 |
| hsa-miR-145-5p | TNIK | -0.82828 | 0.003075 | hsa-miR-145-5p | HECTD1 | 0.744113 | 0.013589 |
| hsa-miR-335-5p | ECT2 | -0.79786 | 0.005677 | hsa-miR-133a-3p | HECTD1 | 0.67533 | 0.032116 |
| hsa-miR-133a-3p | BCL6 | 0.790277 | 0.006514 | hsa-miR-133a-3p | FBLN5 | 0.660606 | 0.044027 |
| hsa-miR-145-5p | BCL6 | 0.83891 | 0.002414 | hsa-miR-145-5p | FBLN5 | 0.684848 | 0.035092 |
| hsa-miR-133a-3p | BCL6 | 0.69697 | 0.031141 | hsa-miR-335-5p | FBLN5 | 0.648485 | 0.049043 |
| hsa-miR-145-5p | BCL6 | 0.769697 | 0.013672 | hsa-miR-335-5p | LGMN | 0.683349 | 0.029377 |
| hsa-miR-224-5p | BCL6 | 0.672727 | 0.039381 | hsa-miR-335-5p | CRIP2 | 0.683349 | 0.029377 |
| hsa-miR-224-5p | UVSSA | -0.74424 | 0.013565 | hsa-miR-224-5p | DIS3L | 0.90044 | 0.000381 |
| hsa-miR-224-5p | WDR19 | -0.68485 | 0.035092 | hsa-miR-145-5p | PML | -0.63382 | 0.049095 |
| hsa-miR-224-5p | CXCL9 | -0.66061 | 0.044027 | hsa-miR-133a-3p | PML | -0.67856 | 0.030994 |
| hsa-miR-145-5p | CXCL9 | -0.73333 | 0.021166 | hsa-miR-335-5p | PML | -0.81278 | 0.004259 |
| hsa-miR-335-5p | CXCL9 | -0.70909 | 0.027514 | hsa-miR-133a-3p | MT1F | 0.854545 | 0.003505 |
| hsa-miR-215-5p | CXCL9 | -0.66061 | 0.044027 | hsa-miR-215-5p | MT1F | 0.648485 | 0.049043 |
| hsa-miR-133a-3p | SYNPO2 | 0.648485 | 0.049043 | hsa-miR-145-5p | MT1F | 0.793939 | 0.009844 |
| hsa-miR-335-5p | CPE | 0.648485 | 0.049043 | hsa-miR-335-5p | MT1F | 0.733333 | 0.021166 |
| hsa-miR-215-5p | ACSL1 | 0.793939 | 0.009844 | hsa-miR-335-5p | PELP1 | 0.700649 | 0.024019 |
| hsa-miR-335-5p | ACSL1 | 0.806061 | 0.008236 | hsa-miR-215-5p | CLDN7 | 0.717624 | 0.019454 |
| hsa-miR-224-5p | ACSL1 | 0.878788 | 0.001977 | hsa-miR-224-5p | CLDN7 | 0.678834 | 0.030899 |
| hsa-miR-224-5p | NPR3 | 0.854545 | 0.003505 | hsa-miR-335-5p | CLDN7 | 0.808135 | 0.00467 |
| hsa-miR-224-5p | IL6ST | 0.805691 | 0.004897 | hsa-miR-145-5p | RASD1 | 0.806061 | 0.008236 |
| hsa-miR-215-5p | IL6ST | 0.798863 | 0.005573 | hsa-miR-133a-3p | RASD1 | 0.745455 | 0.018414 |
| hsa-miR-133a-3p | PDE4D | 0.662617 | 0.036806 | hsa-miR-224-5p | RASD1 | 0.660606 | 0.044027 |
| hsa-miR-133a-3p | PDE4D | 0.828283 | 0.003075 | hsa-miR-224-5p | NSF | -0.69176 | 0.026678 |
| hsa-miR-145-5p | PDE4D | 0.766929 | 0.009638 | hsa-miR-133a-3p | ACSF2 | -0.64182 | 0.04544 |
| hsa-miR-224-5p | ENC1 | -0.74424 | 0.013565 | hsa-miR-335-5p | ACSF2 | -0.66913 | 0.034348 |
| hsa-miR-145-5p | POLK | 0.717329 | 0.019528 | hsa-miR-133a-3p | RPS6KB1 | 0.756415 | 0.011341 |
| hsa-miR-133a-3p | POLK | 0.656538 | 0.039204 | hsa-miR-145-5p | RPS6KB1 | 0.730554 | 0.016409 |
| hsa-miR-335-5p | POLK | 0.711249 | 0.021091 | hsa-miR-224-5p | RPS6KB1 | 0.652973 | 0.040658 |
| hsa-miR-224-5p | POLK | -0.66061 | 0.044027 | hsa-miR-145-5p | PRKAR1A | 0.717845 | 0.019399 |
| hsa-miR-224-5p | PDE8B | 0.894187 | 0.000482 | hsa-miR-133a-3p | PRKAR1A | 0.828283 | 0.003075 |
| hsa-miR-215-5p | PDE8B | 0.725354 | 0.017591 | hsa-miR-335-5p | PRKAR1A | 0.754658 | 0.011645 |
| hsa-miR-145-5p | DCP2 | -0.68335 | 0.029377 | hsa-miR-145-5p | ABCA8 | 0.721212 | 0.024195 |
| hsa-miR-133a-3p | DCP2 | -0.70065 | 0.024019 | hsa-miR-133a-3p | ABCA8 | 0.806061 | 0.008236 |
| hsa-miR-335-5p | DCP2 | -0.68335 | 0.029377 | hsa-miR-133a-3p | CDC42EP4 | 0.745455 | 0.018414 |
| hsa-miR-133a-3p | CCDC69 | 0.898646 | 0.000408 | hsa-miR-224-5p | LLGL2 | -0.81278 | 0.004259 |
| hsa-miR-215-5p | CCDC69 | 0.743484 | 0.013712 | hsa-miR-145-5p | ITGB4 | 0.797863 | 0.005677 |
| hsa-miR-145-5p | CCDC69 | 0.821065 | 0.003592 | hsa-miR-133a-3p | ITGB4 | 0.797863 | 0.005677 |
| hsa-miR-224-5p | CCDC69 | 0.691764 | 0.026678 | hsa-miR-224-5p | UBE2O | -0.65046 | 0.041705 |
| hsa-miR-335-5p | CCDC69 | 0.821065 | 0.003592 | hsa-miR-224-5p | TMC6 | -0.68931 | 0.027446 |
| hsa-miR-133a-3p | NEDD9 | -0.68335 | 0.029377 | hsa-miR-335-5p | PLIN4 | 0.69697 | 0.031141 |
| hsa-miR-145-5p | NEDD9 | -0.70065 | 0.024019 | hsa-miR-215-5p | PLIN4 | 0.890909 | 0.00138 |
| hsa-miR-335-5p | NEDD9 | -0.70065 | 0.024019 | hsa-miR-224-5p | PLIN4 | 0.890909 | 0.00138 |
| hsa-miR-335-5p | CD83 | -0.66061 | 0.044027 | hsa-miR-224-5p | USF2 | -0.67085 | 0.03372 |
| hsa-miR-133a-3p | BTN3A3 | -0.7233 | 0.018074 | hsa-miR-224-5p | DYRK1B | 0.64873 | 0.042436 |
| hsa-miR-145-5p | BTN3A3 | -0.76804 | 0.00947 | hsa-miR-224-5p | LTBP4 | -0.65116 | 0.041412 |
| hsa-miR-224-5p | UBD | -0.7697 | 0.013672 | hsa-miR-133a-3p | ZNF226 | -0.66364 | 0.036412 |
| hsa-miR-145-5p | UBD | -0.67273 | 0.039381 | hsa-miR-145-5p | ZNF226 | -0.76804 | 0.00947 |
| hsa-miR-215-5p | UBD | -0.80606 | 0.008236 | hsa-miR-224-5p | DMPK | -0.72121 | 0.024195 |
| hsa-miR-145-5p | PLA2G7 | -0.70065 | 0.024019 | hsa-miR-145-5p | DMPK | -0.64848 | 0.049043 |
| hsa-miR-133a-3p | PLA2G7 | -0.68335 | 0.029377 | hsa-miR-335-5p | DMPK | -0.72121 | 0.024195 |
| hsa-miR-335-5p | PLA2G7 | -0.70065 | 0.024019 | hsa-miR-133a-3p | ZNF341 | 0.678557 | 0.030994 |
| hsa-miR-224-5p | DST | -0.66061 | 0.044027 | hsa-miR-133a-3p | DSN1 | -0.69347 | 0.026153 |
| hsa-miR-335-5p | DST | -0.69697 | 0.031141 | hsa-miR-335-5p | DSN1 | -0.79786 | 0.005677 |
| hsa-miR-224-5p | ZNF451 | 0.74424 | 0.013565 | hsa-miR-215-5p | SLPI | 0.733333 | 0.021166 |
| hsa-miR-145-5p | TMEM30A | -0.67883 | 0.030899 | hsa-miR-133a-3p | SLPI | 0.818182 | 0.006811 |
| hsa-miR-133a-3p | PERP | 0.674775 | 0.032312 | hsa-miR-224-5p | SLPI | 0.793939 | 0.009844 |
| hsa-miR-145-5p | PERP | 0.70517 | 0.022738 | hsa-miR-145-5p | SLPI | 0.793939 | 0.009844 |
| hsa-miR-224-5p | GPER1 | 0.64873 | 0.042436 | hsa-miR-335-5p | SLPI | 0.854545 | 0.003505 |
| hsa-miR-224-5p | SNX13 | -0.80374 | 0.005084 | hsa-miR-335-5p | FAM65C | 0.672727 | 0.039381 |
| hsa-miR-133a-3p | PDK4 | 0.854545 | 0.003505 | hsa-miR-133a-3p | AGPAT3 | 0.898646 | 0.000408 |
| hsa-miR-335-5p | PDK4 | 0.963636 | 0 | hsa-miR-145-5p | AGPAT3 | 0.782275 | 0.007488 |
| hsa-miR-145-5p | PDK4 | 0.769697 | 0.013672 | hsa-miR-215-5p | AGPAT3 | 0.652973 | 0.040658 |
| hsa-miR-133a-3p | PDK4 | 0.939394 | 0 | hsa-miR-335-5p | AGPAT3 | 0.795205 | 0.005961 |
| hsa-miR-335-5p | PDK4 | 0.830303 | 0.005557 | hsa-miR-224-5p | AGPAT3 | 0.652973 | 0.040658 |
| hsa-miR-145-5p | PDK4 | 0.878788 | 0.001977 | hsa-miR-145-5p | POFUT2 | 0.656491 | 0.039223 |
| hsa-miR-133a-3p | MEST | 0.681033 | 0.030151 | hsa-miR-133a-3p | POFUT2 | 0.791471 | 0.006377 |
| hsa-miR-335-5p | MEST | 0.803741 | 0.005084 | hsa-miR-133a-3p | BCL2L13 | 0.691764 | 0.026678 |
| hsa-miR-215-5p | PLXNA4 | 0.793939 | 0.009844 | hsa-miR-145-5p | BCL2L13 | 0.782275 | 0.007488 |
| hsa-miR-335-5p | PLXNA4 | 0.854545 | 0.003505 | hsa-miR-133a-3p | SLC2A11 | 0.792035 | 0.006313 |
| hsa-miR-224-5p | PLXNA4 | 0.806061 | 0.008236 | hsa-miR-215-5p | EIF4ENIF1 | 0.703272 | 0.02327 |
| hsa-miR-224-5p | TBXAS1 | -0.76804 | 0.00947 | hsa-miR-335-5p | EIF4ENIF1 | 0.648649 | 0.04247 |
| hsa-miR-224-5p | HMBOX1 | 0.64873 | 0.042436 | hsa-miR-224-5p | EIF4ENIF1 | 0.723756 | 0.017965 |
| hsa-miR-335-5p | SFRP1 | 0.721212 | 0.024195 | hsa-miR-215-5p | ODF3B | 0.812777 | 0.004259 |
| hsa-miR-215-5p | FABP4 | 0.806061 | 0.008236 | hsa-miR-133a-3p | ODF3B | 0.753123 | 0.011915 |
| hsa-miR-224-5p | FABP4 | 0.878788 | 0.001977 | hsa-miR-224-5p | ODF3B | 0.753123 | 0.011915 |
| hsa-miR-335-5p | FABP4 | 0.745455 | 0.018414 | hsa-miR-145-5p | ODF3B | 0.633817 | 0.049095 |
| hsa-miR-133a-3p | IL33 | 0.866667 | 0.002681 | hsa-miR-335-5p | ODF3B | 0.723297 | 0.018074 |
| hsa-miR-145-5p | IL33 | 0.733333 | 0.021166 | hsa-miR-145-5p | FHL1 | 0.717329 | 0.019528 |
| hsa-miR-335-5p | IL33 | 0.830303 | 0.005557 | hsa-miR-133a-3p | FHL1 | 0.765961 | 0.009787 |
|  |  |  |  | hsa-miR-224-5p | FHL1 | 0.729487 | 0.016647 |
|  |  |  |  | hsa-miR-215-5p | FHL1 | 0.699091 | 0.024471 |
|  |  |  |  | hsa-miR-335-5p | FHL1 | 0.869305 | 0.001087 |
